# Ultraconserved coding regions outside the homeobox of mammalian Hox genes

**DOI:** 10.1186/1471-2148-8-260

**Published:** 2008-09-24

**Authors:** Zhenguo Lin, Hong Ma, Masatoshi Nei

**Affiliations:** 1 Department of Biology and Institute of Molecular Evolutionary Genetics, Pennsylvania State University, University Park, PA 16802, USA; 2 Huck Institutes of Life Sciences, Pennsylvania State University, University Park, PA 16802, USA

## Abstract

**Background:**

All bilaterian animals share a general genetic framework that controls the formation of their body structures, although their forms are highly diversified. The Hox genes that encode transcription factors play a central role in this framework. All Hox proteins contain a highly conserved homeodomain encoded by the homeobox motif, but the other regions are generally assumed to be less conserved. In this study, we used comparative genomic methods to infer possible functional elements in the coding regions of mammalian Hox genes.

**Results:**

We identified a set of ultraconserved coding regions (UCRs) outside the homeobox of mammalian Hox genes. Here a UCR is defined as a region of at least 120 nucleotides without synonymous and nonsynonymous nucleotide substitutions among different orders of mammals. Further analysis has indicated that these UCRs occur only in placental mammals and they evolved apparently after the split of placental mammals from marsupials. Analysis of human SNP data suggests that these UCRs are maintained by strong purifying selection.

**Conclusion:**

Although mammalian genomes are known to contain ultraconserved non-coding elements (UNEs), this paper seems to be the first to report the UCRs in protein coding genes. The extremely high degree of sequence conservation in non-homeobox regions suggests that they might have important roles for the functions of Hox genes. We speculate that UCRs have some gene regulatory functions possibly in relation to the development of the intra-uterus child-bearing system.

## Background

An unexpected feature of mammalian genomes is that they contain a large number of ultraconserved DNA elements [1]. These elements have been shown to be under strong purifying selection, and therefore they are believed to have some important biological functions [2]. The specific functions of these elements and the mechanism that led to formation of these regions remain unclear. Some studies have suggested that these regions may play a role in the regulation of their neighboring developmental genes [3,4]. These ultraconserved elements have been identified almost exclusively from noncoding regions of the genome.

During the course of studying DNA sequence divergence of Hox genes among different mammalian orders, we noticed that many ultraconserved regions exist in the protein coding regions outside the homeobox. Hox genes encode a group of transcription factors that control the segmentation identities of developing animal embryos along the head-to-tail axis. These proteins contain a domain called the homeodomain encoded by the homeobox motif. The amino acid sequences of the homeodomain have been conserved even between mammals and insects [5]. At the nucleotide level, however, synonymous nucleotide substitutions occur with reasonably high frequencies [6]. Therefore, the homeobox motifs are not ultraconserved regions.

**Figure 1:**
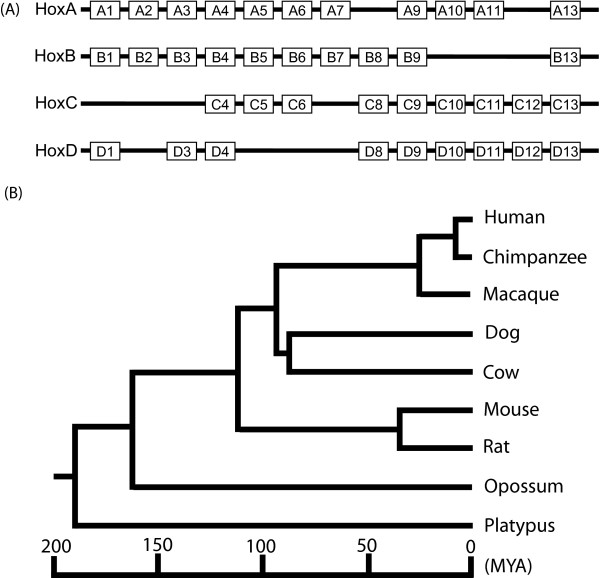
**A. Diagram showing the chromosomal organization of Hox genes in human.** Each horizontal thick line represents a gene cluster, with cluster name shown at the left side. Clusters are shown from 3' end to 5' end. The 13 cognate gene groups are defined vertically across the clusters and consisting of up to four genes, each on a different chromosome. Some genes in each cognate group have been lost during evolution. B. A phylogenetic tree illustrating the evolutionary relationships and approximate divergence times of representative mammalian species. The branch lengths represent the estimated divergence times. The divergence times were obtained from the TimeTree database[50]. MYA: million years ago.

Hox genes tend to be organized into gene clusters, and there is a striking correlation between the order of Hox genes in the cluster and the spatial patterns of their expression in the developing embryo. The Hox genes at the 3' end of the cluster are expressed in the anterior regions of the embryo and those genes at the 5'end are expressed in the posterior regions. This cluster organization and the expression pattern of Hox genes are highly conserved from arthropods to mammals [7]. Multiple duplication events of these gene clusters have led to a significant expansion of the Hox gene family in vertebrates. As a result, four Hox clusters (denoted as HoxA, HoxB, HoxC and HoxD) are present on separate chromosomes in mammalian species. According to the position in the clusters, the Hox genes in the four clusters can be classified into thirteen cognate (orthologous gene) groups. However, some members of these cognate groups have been lost, and only 39 Hox genes are currently present in the human and other mammalian genomes (Fig. 1A).

Although the homeodomain is highly conserved, the sequences outside the homeodomain in Hox proteins are generally quite divergent and do not contain conserved domains except some small motifs such as the MXSXFE motif at the N-terminus and the YPWM motif near the homeodomain. Some studies have been conducted on these non-homeodomain regions of *Drosophila *Hox genes. For example, the C-terminal region of the *Drosophila Ubx *protein was shown to serve as a repressor domain, and sequence changes in this region are correlated with limb pattern differences between insects and crustaceans [8,9]. Another study on the *Drosophila Abd-B *gene indicates that protein domains outside the homeodomain influence the activation or repression of target gene expression [10]. These studies suggest that protein regions outside the homeodomain of other Hox proteins might also have some effects on embryonic development and morphological evolution. In this study, we examined the rates of nucleotide substitutions in different coding regions of Hox genes to identify potentially important functional elements. Interestingly, we found that many ultraconserved regions are present between orthologous mammalian Hox genes, and most of them are located outside the homeobox motif, indicating that they are probably important for the functions of Hox genes.

## Results and discussion

### Ultraconserved Coding Regions (UCRs) present in many mammalian Hox genes

**Figure 2:**
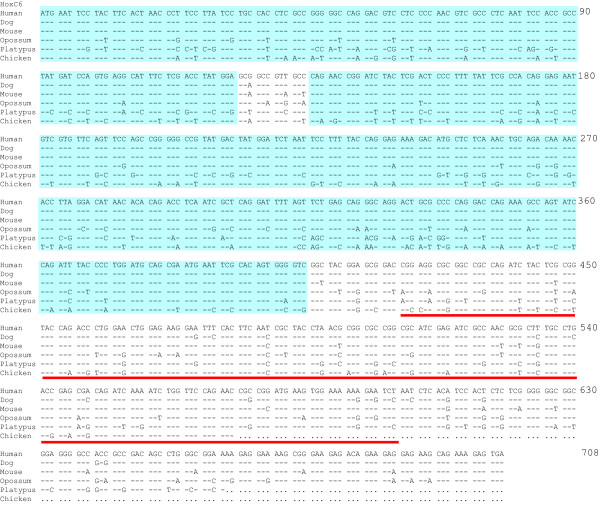
**Nucleotide sequences of orthologous HoxC6 genes from six mammalian species**. The ultraconserved coding regions (UCRs) are shaded by blue and the homeobox motif is underlined in red.

**Figure 3:**
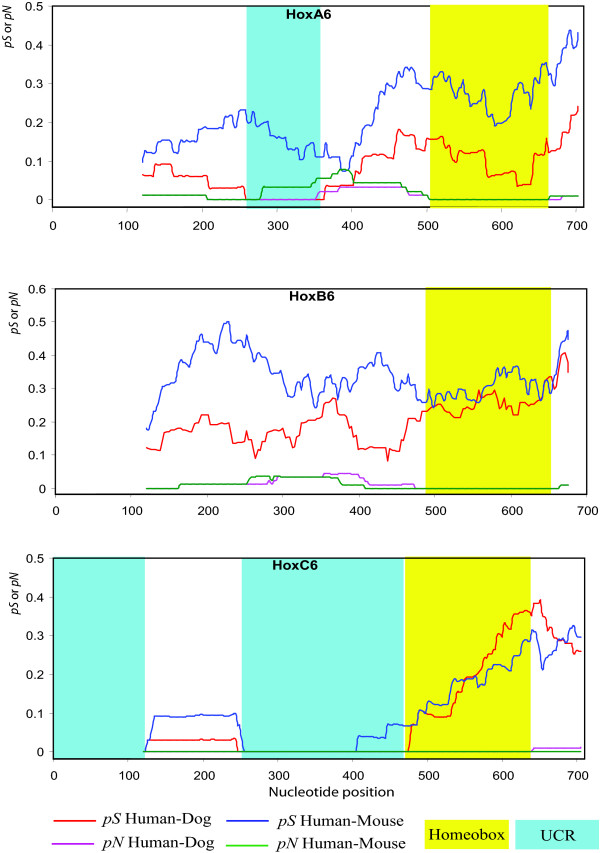
**Sliding window analysis of synonymous and nonsynonymous nucleotide substitutions between human and dog or human and mouse for the orthologous gene group 6**. The window size is 120 nucleotides and moves every single codon. The X axis indicates the position of the last nucleotide of each sliding window comparison. The regions of homeobox motif and UCRs are highlighted in yellow and light blue, respectively. The synonymous and nonsynonymous substitutions are measured by the proportion of synonymous differences per synonymous site (*pS*), and the proportion of nonsynonymous differences per nonsynonymous site (*pN*), respectively. We used *pS *and *pN *here because these measures are model free and range from 0 to 1 (see Nei and Kumar 2000).

A preliminary sliding window analysis was performed to study the synonymous and nonsynonymous nucleotide substitutions between orthologous Hox genes from human, dog and mouse to identify conserved coding regions. We then found that some coding regions are ultraconserved between different species. Neither nonsynonymous nor synonymous substitutions were observed in these regions, which may include as many as hundreds of nucleotide sites (Fig. 2 and 3). Because these species have diverged over 90 million years ago [11,12], it is unlikely to observe these conserved regions if the synonymous substitutions are free from natural selection [13]. Considering that the nucleotide identity between the human and mouse genomes is about 40% [14], the probability of observing two identical sequences with more than 100 consecutive nucleotide sites by chance will be = (0.4)^-100^. Therefore, it appears that the synonymous sites as well as the nonsynonymous sites in these regions have been highly conserved.

In the present study, we defined an Ultraconserved Coding Region (UCR) as a region with at least 120 nucleotides (40 codons) that do not contain any synonymous substitutions between two distantly related species (divergence time ~90 million years or more). Using 120 nucleotides as the threshold is not based on considerations of biological functions but merely statistical convenience. It can be shown that the probability of occurrence of a UCR by chance is extremely small if this criterion is applied.

To detect the UCRs in the mammalian lineages, we performed a sliding window analysis of synonymous and nonsynonymous nucleotide substitutions for each pair of Hox orthologous genes from six species (human, dog, cow, mouse, opossum and platypus) representing six different mammalian orders (Fig 1B). We found that the UCRs in Hox genes are frequently detected in placental mammals (Table. 1 and Additional file 1). For example, 33 UCRs were detected in 21 Hox genes in the dog/cow comparison (Table 1). We also found 26 UCRs in 19 Hox orthologous genes between human and dog, 23 UCRs in 19 Hox genes in the human/cow comparison, and 14 UCRs in 13 Hox genes in the human/mouse comparison. In summary, at least 12 UCRs were found in the Hox genes in each pairwise comparison of placental mammals. Furthermore, many of these UCRs were found repeatedly in different pairwise comparisons (see Additional file 1), indicating that these regions have been conserved throughout the evolution of placental mammals. Considering that the divergence time between dog and cow is less than 90 million years and their Hox sequences are highly similar, we decided not to include the UCRs that are only found in the two species for further analysis.

**Table 1:** List of numbers of UCRs identified in the mammalian Hox genes

	Dog	Cow	Mouse	Opossum	Platypus
Human	19/26	19/23	13/14	2/2	0
Dog	-	21/33	10/12	2/2	0
Cow		-	14/16	2/2	0
Mouse			-	2/2	0
Opossum				-	0

Notes: The first number represents the number of Hox genes that contain UCRs. The second number refers to the total number of UCRs identified in the pairwise comparison between two species.

In contrast, the number of UCRs is significantly reduced in the comparison between a placental mammal and the marsupial opossum (*Monodelphis domestica*) or duck-billed platypus (*Ornithorhynchus anatinus*). This paucity of UCRs could possibly be due to longer divergence times between these species, and therefore more synonymous substitutions have accumulated. Although another possible reason is that the coverage of genomic sequences of opossum and platypus are incomplete and several Hox genes were not retrieved, this is unlikely to affect our results because we did uncover most of the Hox genes from these species and only about half of the missing ones have UCRs in comparison of genes from placental mammals. Similarly, UCRs were not detected in Hox orthologs between two closely related puffer fishes, *Takifugu rubripes *and *Tetraodon nigroviridis*, even though the time of divergence between these two species has been estimated to be only 18–30 million years [15]. Furthermore, we did not identify any UCR for 8 Hox gene pairs between two *Drosophila *species, *D. melanogaster *and *D. pseudoobscura*, whose divergence time is less than 30 million years [16]. Therefore, the presence of UCRs in the Hox genes is likely to be unique to placental mammals.

Although we used a threshold of 120 nucleotides, the length of a UCR was often much longer than this. For example, a UCR of 348 nucleotides is present in HoxC6 genes between the human and dog, and this region is also highly conserved in all other placental mammals (Fig. 2). Note that this region is located outside the highly conserved homeobox motif. In the homeobox motif, a number of synonymous substitutions were observed although the nonsynonymous sites are identical (Fig. 3). The difference of the conservation level of synonymous sites between the homeobox and UCR regions indicates that the occurrence of UCRs is more likely to be due to purifying selection at both synonymous and nonsynonymous sites. It also indicates that the appearance of UCRs is unlikely to be due to conservation of protein sequence, although it was previously found that highly conserved nonsynonymous sites are usually associated with conserved synonymous sites [17]. The mammalian Hox genes usually contain two exons and one intron. The homeobox motif is located in the second exon in all mammalian Hox genes. However, most UCRs are found in the first exon, and many of them appear at the 5'end of the coding sequences (CDS) (see Additional file 1), indicating that they are located in non-homeobox regions.

Although a long UCR is present in HoxC6 gene, the other Hox6 group genes do not contain such a long UCR. For example, many synonymous substitutions are detected in the corresponding region of HoxB6 (Fig. 3). Such differences were also observed between members of other cognate groups (see Additional file 1). In addition, even if UCRs are present in all genes of a cognate group (e.g., group 5 and 8), UCRs are observed at somewhat different locations (see Additional file 1).

Comparison of the frequency of UCRs among different gene clusters and different regions of clusters showed that the frequency varies significantly with gene pair. First, UCRs are more frequent in the HoxC cluster than in any other clusters. Ten UCRs are detected for 8 out of 9 HoxC cluster genes, and HoxC genes usually contain long UCRs. In contrast, only 20 UCRs are found in the other three gene clusters (30 genes). Second, more UCRs are present in the central cognate groups (Hox4-8) than those genes of anterior (Hox1-3) and posterior groups (Hox9-13). For example, 11 out of 15 central Hox genes contain at least one UCR (total = 15), while only 11 UCRs were detected among 24 Hox genes of the other two groups. Furthermore, there is a significant difference in the fraction of DNA sequence involved in UCRs between central and non-central Hox genes. The UCRs in the central group of genes contain 3684 nucleotides, accounting for 32% of coding sequences, whereas only 10% of nucleotides are involved in UCR sequences in the other groups of Hox genes.

### Origin and evolution of UCRs

**Figure 4:**
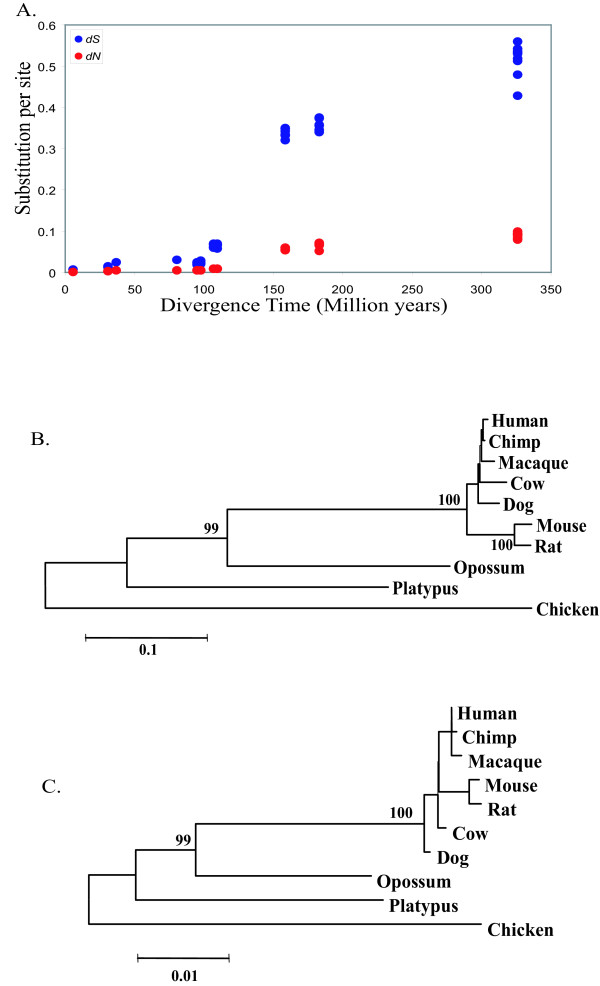
**Non-linear accumulation of synonymous substitutions in the Hox UCRs**. **A**. The numbers of synonymous and nonsynonymous substitutions between two species are plotted against their divergence times, which were obtained from the TimeTree database[50]. **B**. Neighbor-Joining trees based on synonymous substitutions (*d*_*S*_) which were computed by the pairwise deletion option (Nei and Kumar 2000). The chicken sequence was used as the outgroup. This tree shows short branches for placental mammals which diverged during the last 100 million years. Opossum and placental mammals have diverged about 145 MYA, but the branch length for opossum is shorter than that for placental mammals. This result indicates that the *d*_*S *_for placental mammals increased rather rapidly until their emergence. C. Neighbor-Joining tree for *d*_*N*_. The evolutionary pattern of this tree is similar to that of the tree for *d*_*S*_.

To study the origin and evolution of the UCRs, we concatenated the nucleotide sequences of 32 UCRs which were shared by 22 mammalian Hox sequences (see Additional file 2) for the seven eutherian species. We also concatenated the corresponding UCR region of the Hox genes from opossum, platypus and chicken, and these sequences were aligned with the eutherian sequences (The multiple sequence alignments are available in Additional file 3). We then computed the numbers of synonymous (*d*_*S*_) and nonsynonymous (*d*_*N*_) substitutions for all pairs of ten species. We then related the *d*_*S *_and *d*_*N *_values for a pair species to their divergence time. The results of the study are presented in Fig. 4A. This figure shows that both *d*_*S *_and *d*_*N *_do not increase appreciably for the first 100 million years during which placental mammals (or eutherians) evolved. As the divergence time increased beyond 100 million years, however, *d*_*S *_rapidly increased up to 340 million years ago (MYA) when eutherians and chicken diverged. The rate of increase of *d*_*N *_was slow beyond the first 100 million years but steadily increased up to 340 MYA. These results clearly showed that the eutherian UCRs evolved around the time of origin of placental mammals. This conclusion is consistent with previous observation that UCRs exist primarily between eutherian genomes.

To clarify this situation graphically, we constructed the Neighbor-Joining tree of the species involved using the *d*_*S *_and *d*_*N *_values with the chicken sequence as the outgroup (Fig. 4B, C). Fig. 4B shows that in the eutherian lineage *d*_*S *_increased relatively rapidly before the eutherian radiation but virtually stopped increasing thereafter. This again supports the evolution of UCRs in the early stage of eutherian evolution. A similar result was observed by *d*_*N*_, though the reliability of estimates of branch lengths is low in this case because of the small *d*_*N *_values.

Why did UCRs evolved in placental mammals and how are they conserved in the genome? It is very difficult to answer these questions at the present. However, we noticed that the origin of placental mammals depended on the evolution of placenta, which grows inside the mother's uterus and functions as a way to exchange gas and nutrients between the mother and fetus during gestation [18]. It has been suggested that the placenta of eutherian mammals evolved from a much simpler tissue attached to the eggshell of birds and reptiles [18]. Many Hox genes, including HoxA4, HoxA7, HoxA11, HoxC4, HoxC5, HoxC6, and HoxC8, have been shown to be expressed during placental development [19-21]. It is interesting to note that most of these Hox genes are located in the central regions of Hox gene clusters and contain at least one UCR in the coding regions. Although the specific functions of these Hox genes in placental development are still unclear, these observations suggest that they are involved in the growth and differentiation of trophoblasts. Considering that the Hox genes play important roles in the development of animal embryo development, we can postulate that the eutherian UCRs might have developed in association with the evolution of placentae. According to this hypothesis, we could further speculate that new mutations in the UCRs could have been deleterious after the formation of placentae and these mutations have been eliminated by purifying selection. In summary, the UCR sequence might be important for the function of Hox genes in formation of the placenta and have contributed to the evolution of placental mammals.

### Mutation rate and the maintenance of UCRs

One of the possible explanations of the maintenance of UCRs for a long evolutionary time in the eutherian genome could be a low mutation rate that might be observed in this specific genomic region. If this hypothesis is correct, one would expect that the rate of nucleotide substitution is lower in the intronic and intergenic regions as well as in the UCRs in these Hox gene regions than in other regions. Actually, there is some evidence that the mutation rate is not constant throughout the genome but varies with genomic region in mammals [14,22]. We therefore tested this hypothesis by computing the rate of nucleotide substitution in the intronic and intergenic regions of the Hox gene complex as well as the rate of synonymous substitution. We studied this problem in relation to the locations of Hox genes because the genes located in the central region of the Hox gene complex contain UCRs more often than the genes in the noncentral regions.

**Figure 5:**
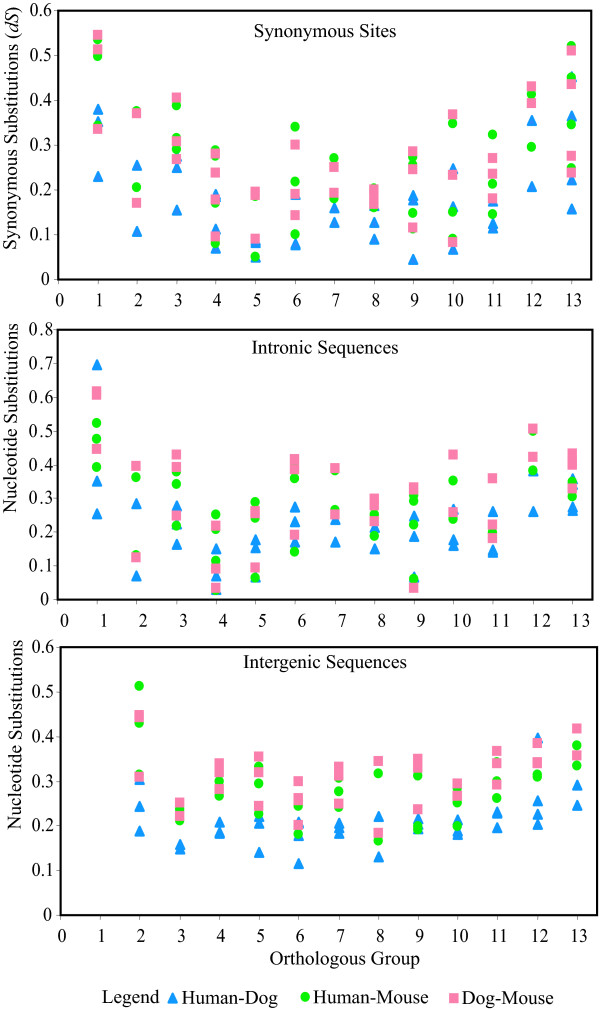
**Reduced substitution rates in the central region of Hox gene clusters**. **A**. Correlation between the synonymous mutation rates (*d*_*S*_) of coding regions of Hox genes and their locations in the Hox cluster. The *d*_*S *_values are plotted against the corresponding number of cognate groups. **B**. Correlation between nucleotide substitution rates of intronic sequences of each Hox genes and their positions on the cluster. **C**. Correlation between nucleotide substitution rates of intergenic sequences and their positions on the Hox cluster. The numbers on the x axis represent the numbers of cognate group at 5' flanking end of each intergenic sequence.

The rate of nucleotide substitution between a pair of species was measured by the number of nucleotide substitution (*d*) between them by using the Jukes-Cantor formation [23]. Similarly, the rate of synonymous substitution was measured by the number of synonymous nucleotide substitutions (*d*_*S*_) using the modified Nei and Gojobori method [23]. The values were computed for all pairs of human, dog, and mouse and opossum. The results obtained are presented in Fig 5. Fig. 5A shows that the *d*_*S *_values are significantly correlated with the positions of the genes in the Hox gene cluster. The relationship is more or less U-shaped, and the Hox genes at both ends of the cluster (e.g., cognate groups 1 and 13) have higher *d*_*S *_than the genes in the central region (cognate groups 4–8). The *d*_*S *_values of central cognate genes are significantly lower than those of other Hox genes (*P *< 3.47 × 10^-7^). This pattern of *d*_*S *_values was not observed in the comparison of Hox genes between the two closely related puffer fishes and between *D. melanogaster *and *D. pseudoobscura*. Therefore, it is likely that only mammalian Hox gene clusters show such a position effect, which is consistent with the occurrence of UCRs only in eutherians.

In addition, the numbers of nucleotide substitutions (*d*) of intronic sequences in Hox genes shows a similar U-shaped curve (Fig. 5B), and these values showed a significant correlation with the *d*_*S *_values (*R *= 0.6465, *P *< 1.488 × 10^-5^). Similarly, the *d *values for the intergenic sequences also showed a U-shape pattern (Fig. 5C). Therefore, all sites in the central regions of Hox gene clusters (including coding, intronic and intergenic regions) are more conserved than those of the anterior or posterior genes. This suggests that low mutation rate in the central region of the Hox gene cluster is one factor for generating the higher frequency of UCRs in this region. However, the *d*_*S *_values in the central region of Hox gene complex was significantly lower than the *d *values for the intronic and intergenic region (*P *< 1.29 × 10^-4^). This higher degree of conservation of the synonymous sites suggests that purifying selection is a more important factor for generating UCRs than the lower mutation rate.

### UCRs have reduced density of synonymous SNPs and nucleotide diversity

The highly conserved synonymous sites of the UCRs indicate that most mutations at these sites should have some deleterious effects and purifying selection eliminate the mutations. Therefore, if the conservation of UCRs is due to purifying selection, we would expect a decreased frequency of synonymous Single Nucleotide Polymorphisms (SNPs) and reduced gene diversity (heterozygosity) in the UCRs in the human population. To test this hypothesis, we used human SNP data to estimate the frequency of SNPs inside and outside the UCRs. As expected, the frequency of synonymous SNPs in UCRs (2.88/kb) was significantly lower than that in the non-UCRs regions (9.04) of human Hox genes (*P *< 0.01). The frequency of nonsynonymous SNPs within UCRs was 1.44/kb, which is also lower than that of non-UCRs (2.73/kb), although the difference was not significant (*P *= 0.34). The significantly reduced frequency of synonymous SNPs in the UCRs further supports the hypothesis that the synonymous sites in the UCR regions have been constrained by purifying selection.

We also compared the nucleotide diversity (*π*) between UCR and non-UCR regions based on SNP data and observed a significant difference between them. For the synonymous sites, *π *= 6.01 × 10^-4 ^in the UCR regions, compared to *π *= 0.017 in the non-UCR regions. The *π *= 1.9 × 10^-4 ^for nonsynonymous sites in the UCRs, but 4.17 × 10^-4 ^in the non-UCR sequences. Therefore, the UCR regions have much lower nucleotide diversity at both synonymous and nonsynonymous sites than those of non-UCRs. The reduction of nucleotide diversity in the UCRs further supports the idea that population frequencies of deleterious SNP alleles has been reduced by purifying selection. The evolutionary conservation of UCRs, in turn, suggests that the UCR sequences may be important for the proper function of Hox genes.

### UCRs might overlap with novel transcripts or function as regulatory elements

Recent studies have shown that synonymous sites could be affected by natural selection [24-28]. For example, the synonymous codons of many highly expressed genes are not randomly used in many organisms such as bacteria, plants, fungi and invertebrates [29-31]. Moreover, the synonymous sites in the Exonic Splicing Enhancers (ESEs), which affect splicing of pre-mRNA, are also under purifying selection [27,32-34]. However, we did not detect a significant correlation between codon usage and occurrence of UCRs, and there is no statistically significant difference in the density of ESEs between UCRs and other regions (data not shown).

In this study, we found that many UCRs are located either at the 5'end or 3'end of coding regions of genes and their flanking noncoding regions are also highly conserved in some cases. As a consequence, large conserved blocks covering both coding and noncoding regions are present in some Hox genes. For example, the noncoding sequences are highly conserved near the UCRs in HoxC4, HoxC5 and HoxC6 (Additional file 4A), forming long conserved blocks (over 500 nucleotides). Further analysis indicated that these regions are only conserved in placental mammals, similar to the UCRs (Additional file 4B). Currently, information about the functions and evolutionary origins of these conserved blocks is not available. At present time, we can not exclude the possibility that these blocks might be the overlapping regions of Hox genes and other unknown RNA transcript genes.

Such potential overlapping genes can be transcribed in the same or reverse direction to the Hox genes. Antisense RNA transcription has been implicated in various forms of gene regulation, including RNAi-like degradation of corresponding sense RNA transcripts and competition with sense transcription. For example, a 177-nt antisense RNA is transcribed from the central coding region of the photosynthesis gene *IsiA *in cyanobacteria and suppresses the expression of gene *IsiA *under some conditions [35]. A recent study has shown that the HoxA clusters are enriched in antisense transcripts and many of these transcripts overlap with coding regions [36]. Among them, the *HoxA11 *antisense RNA transcript has been well characterized and shown to have a function in regulating the expression of the *HoxA11 *gene [37,38]. We noticed that a *HoxA11 *antisense transcript overlaps with the coding region of the first HoxA11 exon [37], where a UCR is identified in this study. Although we did not find any information in the literature for other antisense transcripts that overlap with UCRs studying Hox genes, the possibility of antisense transcription still remains. A recent detailed analysis on 1% of the human genome indicates that substantial portions of the unannotated sequences are transcribed and many transcripts overlap one another extensively [39]. Therefore, we should consider the possibility that the occurrence of UCRs in the mammalian Hox genes is related to the presence of overlapping RNA transcripts, including antisense RNAs.

Some ultraconserved elements in the noncoding regions of mammalian genomes have been shown to function as regulatory elements of neighboring developmental genes [3,4]. The Hox genes are the master control genes for animal embryonic development and are organized into conserved clusters. There is evidence that this organization is critical for the control of the proper spatial and temporal expression of Hox genes [40]. A number of conserved noncoding sequences of 60 to hundreds of nucleotides have been identified in the intergenic regions of Hox clusters by comparative genomic studies, and they were considered to be putative regulatory elements of Hox genes [41]. Although the UCRs are found in the coding regions, it is possible that these regions contain regulatory elements for their own genes or downstream genes. A similar scenario has been observed about the expression regulation of *β*-globin gene cluster, which has been the subject of extensive studies [42]. Different *β*-globin genes are expressed at various development stages. The elements close to the globin genes and the arrangement of the globin genes control their expression switching in different developmental stages[42]. The Hox genes have a similar cluster organization and they are also expressed differently at various developmental stages. Therefore, the UCRs might also serve as regulatory elements to control their neighboring Hox genes. If this is the case, selection on both regulatory elements and coding sequences in the same region has led to the formation and/or maintenance of these UCRs. Because the UCRs are much longer than usual protein binding motifs, it is possible that multiple binding motifs are present in a single UCR. The sequence, number and order of these motifs could be important for their regulatory functions, and the combination of these factors might explain the formation of the UCRs [42]. Alternatively, these extraordinarily long UCRs might interact with RNAs or DNAs in other genomic regions and such interactions might require more highly conserved sequences. Therefore, it is reasonable to postulate that these UCRs are involved in the regulation of expression of Hox genes.

## Conclusion

It appears that ultraconserved elements are frequently present in the mammalian genomes, especially near or in regulatory genes [1,28]. The appearance of UCRs in mammalian Hox genes suggests that they might play important roles during embryo development. Placental mammals are fundamentally different from other mammals in regard to the early developmental environment and fetal nourishment. Previous studies have shown that a number of Hox genes that contain UCRs are expressed in the placenta, although their functions are still unclear [19-21]. Taking into account the important roles of the Hox genes in animal embryonic development and morphological evolution, we cannot ignore the potential connection between the appearance of UCRs and advent of long gestation periods in placental mammals. Therefore, it would be particularly important to study the functions of the UCRs during fetal development. Point mutations at the synonymous sites in the UCRs could be introduced to test if these sites are required for the proper function of mammalian Hox genes. The experimental test could also provide further understanding about the functions of sequences outside the homeobox motif and their roles in the evolution of placental mammals.

## Experimental procedures

### Data mining

The protein coding sequences (CDS) and genomic sequences of Hox genes of human (*Homo sapiens*), mouse (*Mus musculus*), dog (*Canis familiaris*) and the fruitfly *Drosophila melanogaster *were obtained from the NCBI database. These Hox protein sequences were used as queries to blast the NCBI *nr *database for Hox genes from other representative organisms. TBLASTN was performed against the genomic sequences from NCBI genomic databases to obtain as many Hox sequences as possible from the following species: chimpanzee (*Pan troglodytes*), rhesus macaque (*Macaca mulatta*), cow (*Bos Taurus*), dog (*Canis familiaris*), rat (*Rattus norvegicus*), gray short-tailed opossum (*Monodelphis domestica*), duck-billed platypus (*Ornithorhynchus anatinus*), chicken (*Gallus gallus*), zebrafish (*Danio rerio*), Japanese pufferfish (*Takifugu rubripes*), and green pufferfish (*Tetraodon nigroviridis*). The putative coding regions and their protein products were predicted from their genomic sequences based on sequence homology.

### Sequence analysis

The protein sequences of each Hox orthologous group from human, dog, cow, mouse, opossum and platypus were used to generate a multiple sequence alignment by using ClustalX 1.83 [43]. Corresponding nucleotide sequence alignments were then reexamined to improve the alignments based on these protein sequence alignments to avoid frame-shift errors in GeneDoc [44]. The alignments of all available Hox genes (43 pairs) from Japanese pufferfish and green pufferfish were generated separately using the same procedure. Similarly, the alignments of eight Hox genes from two *Drosophila *species, *D. melanogaster *and *D. pseudoobscura*, were also produced. In order to detect a coding region without any synonymous substitution in a region of 120 nucleotides or longer, a sliding window analysis was performed. The size of each sliding window was 120 nucleotides (40 codons), and it moved by every single codon. In each window, the proportion of synonymous differences per synonymous site (*p*_*S*_) and proportion of nonsynonymous differences per nonsynonymous site (*p*_*N*_) were calculated by using the Nei and Gojobori method to minimize the estimation error [45,46]. The ultraconserved coding region (UCR) was detected if *p*_*S *_= 0 and *p*_*N *_= 0 in these sliding windows. The *perl *script for sliding window analysis was provided by Masafumi Nozawa (Personal communication). The multiple sequence alignment of each UCR was then further inspected to detect the presence of insertions and deletions (indels). Only UCRs without any indel were used in this study.

Multiple nucleotide sequence alignments of the UCRs were constructed using sequences from human, chimpanzee, macaque, dog, mouse, rat, opossum, platypus and chicken. A concatenated alignment was generated by joining all the UCRs. The number of synonymous substitutions per synonymous site (*d*_*S*_) and the number of nonsynonymous substitutions per nonsynonymous site (*d*_*N*_) for each pair of concatenated UCR sequences were estimated by using the modified Nei-Gojobori method in MEGA4 [6,45,47]. Neighbor Joining (NJ) trees of the concatenated UCR sequences were reconstructed separately based on their synonymous and nonsynonymous substitutions in MEGA4.

Pairwise *d*_*S *_values of whole coding regions of Hox genes between human, dog and mouse were calculated by the modified Nei-Gojobori method in MEGA4. The intron and intergenic sequences of Hox clusters were retrieved from human (build 36.2), mouse (Build 37.1) and dog genomic sequences and aligned by ClustalX. Pairwise rates of nucleotide substitutions of these intronic and intergenic sequences were estimated by the same method between the three species.

### SNP, ESE, and Codon usage bias analysis

Single Nucleotide Polymorphisms (SNPs) data for each human Hox gene were obtained from the NCBI dbSNP database. The numbers of polymorphic synonymous and nonsynonymous per site were determined for UCR and non-UCR regions. The nucleotide diversity (average number of nucleotide differences per site between two sequences) was estimated by $\hat{\pi }=\frac{n}{n-1}\sum_{ij}{\hat{x}}_{i}{\hat{x}}_{j}\pi_{ij}$[23], where *n *is the number of DNA sequences examined, and ${\hat{x}}_{i}$ is the population frequency of the *i*-th allele, and *π*_*ij *_is the proportion of different nucleotides between the *i*-th and *j*-th type of DNA sequences. The numbers of detected Exonic Splicing Enhancers (ESEs) in UCRs and non-UCRs of each human Hox gene were obtained by examining the human RESCUE-ESE WebServer [48]. The values of Effective Number of Codons of the Hox genes from different lineages were estimated using the web server of CodonW [49].

## Authors' contributions

ZL and MN designed and conducted data analysis. ZL, MN and HM wrote the manuscript.

## Supplementary Material

Additional file 1 A complete list of identified UCRs based on pairwise comparisons among mammalian Hox genes. Click here for file

Additional file 2 **A list of UCRs of Hox genes that are used for concatenated multiple sequence alignment and phylogenetic tree construction**. The nucleotide positions of each UCR are listed in the right column. The name of each UCR is denoted in parentheses. Click here for file

Additional file 3 Multiple alignments of the nucleotide sequences of UCRs Click here for file

Additional file 4 **Conserved noncoding sequences flanking the UCRs**. **A**. The conservation of noncoding sequences flanking the first exons of HoxC4, HoxC5 and HoxC6 from UCSC Human Genome Brower. The position of the coding regions is highlighted by red bar. Transcription direction is indicated by arrow. **B**. The accumulations of nucleotide mutations in the conserved regions with divergence times of the three Hox genes. The number of substitution per site was estimated using the Jukes-Cantor's method in MEGA4 on the coding region and flanking conserved noncoding sequences of HoxC4, HoxC5 and HoxC6. Click here for file
