# Supplementary material for: Ultraconserved coding regions outside the homeobox of mammalian Hox genes
Source: BMC Evol Biol. 2008 Sep 24;8:260. doi: 10.1186/1471-2148-8-260 (PMC2566984; doi:10.1186/1471-2148-8-260)
Supplement: Additional file 1 — A complete list of identified UCRs based on pairwise comparisons among mammalian Hox genes. [file 1471-2148-8-260-S1.pdf]

Additional file 1. A complete list of identified UCRs based on pairwise comparisons among mammalian Hox genes.

	Human/ Dog	Human/ Cow	Human/ Mouse	Dog /Cow	Dog/ Mouse	Cow/ Mouse	Human/ Opossum	Human/ Platypus	Opossum/ Platypus
**A1**				409-591					
**A2**	1-180	1-180	1-180	1-261	1-243	1-243	43-165		
				268-441					
				754-936					
**A3**									
**A4**									
**A5**	1-174	1-198		1-174					
				178-300					
				325-585	316-459	325-459			
**A6**	139-363			406-558					
**A7**									
**A9**		352-504		415-579					
**A10**				1021-1185					
**A11**	244-363	196-318	106-279	322-495					
**A13**	808-930		1-120						
**B1**									
**B2**	49-225	49-225		1-270	1-144	1-144			
**B3**									
**B4**		331-456	385-507	1-174					
**B5**	1-264	1-156		1-228	1-156	1-156			
				301-432					
**B6**									
**B7**	82-204	82-231	n/a	82-204					
	235-399								
**B8**	376-516	1-135	1-150	577-702		1-135			
	547-684	373-516		376-543					
**B9**	109-237			391-525					
	529-735	442-735		529-756					
**B13**									
**C4**	1-186	1-222	1-192	1-186	1-186	1-192			
		226-363	502-621	313-609					
**C5**	1-120	1-234	1-285	1-120	1-285	1-234			
	193-315	385-507				319-459			
**C6**	1-123	202-408	136-405	202-408	1-129	202-405			
	127-474				136-405				
**C8**	307-444	238-444	238-390	172-303		229-390			
				307-531					
				535-654					
**C9**	n/a	232-381	238-357	n/a	n/a	238-357			
**C10**	745-1029	748-1029	748-1029	745-1029	748-1029	748-1029			
**C11**	736-864	673-798		655-798		640-768			
**C12**	730-852								
