# Supplementary material for: Ultraconserved coding regions outside the homeobox of mammalian Hox genes
Source: BMC Evol Biol. 2008 Sep 24;8:260. doi: 10.1186/1471-2148-8-260 (PMC2566984; doi:10.1186/1471-2148-8-260)
Supplement: Additional file 2 — A list of UCRs of Hox genes that are used for concatenated multiple sequence alignment and phylogenetic tree construction. The nucleotide positions of each UCR are listed in the right column. The name of each UCR is denoted in parentheses. [file 1471-2148-8-260-S2.pdf]

Additional file 2. A list of UCR regions of Hox genes that are used for concatenated multiple sequence alignment and phylogenetic tree construction. The nucleotide positions of each UCR are listed in the right column. The name of each UCR is denoted in parentheses.

Gene Name	Nucleotide Positions of UCR (Name)		
HoxA2	1-243 (A2)		
HoxA5	1-198 (A5-1)	316-459(A5-2)	
HoxA6	139-363 (A6)		
HoxA9	352-504 (A9)		
HoxA11	106-363 (A11)		
HoxA13	1-120 (A13-1)	808-930 (A13-2)	
HoxB2	1-225 (B2)		
HoxB4	331-507 (B4)		
HoxB5	1-264 (B5)		
HoxB7	82-231 (B7-1)	235-399 (B7-2)	
HoxB8	1-150 (B8-1)	373-516 (B8-2)	547-684 (B8-3)
HoxB9	109-237 (B9-1)	442-735 (B9-2)	
HoxC4	1-222 (C4-1)	226-363 (C4-2)	502-621 (C4-3)
HoxC5	1-507 (C5)		
HoxC6	1-474 (C6)		
HoxC8	229-444 (C8)		
HoxC9	232-381 (C9)		
HoxC10	745-1029 (C10)		
HoxC11	640-864 (C11)		
HoxC12	730-852 (C12)		
HoxD8	376-612 (D8)		
HoxD10	1-246 (D10-1)	250-372 (D10-2)	742-1011 (D10-3)
