# Supplementary material for: Ultraconserved coding regions outside the homeobox of mammalian Hox genes
Source: BMC Evol Biol. 2008 Sep 24;8:260. doi: 10.1186/1471-2148-8-260 (PMC2566984; doi:10.1186/1471-2148-8-260)
Supplement: Additional file 3 — Multiple alignments of the nucleotide sequences of UCRs [file 1471-2148-8-260-S3.pdf]

Additional file 3. Multiple alignments of the nucleotide sequences of UCRs

Index

Number	UCR Name	Page Number
1	A2	2
2	A5-1	3
3	A5-2	4
4	A6	5
5	A9	6
6	A11	7
7	A13-1	8
8	A13-2	9
9	B2	10
10	B4	11
11	B5	12
12	B7-1	13
13	B7-2	14
14	B8-1	15
15	B8-2	16
16	B8-3	17
17	B9-1	18
18	B9-2	19
19	C4-1	20
20	C4-2	21
21	C4-3	22
22	C5	23
23	C6	25
24	C8	27
25	C9	28
26	C10	29
27	C11	30
28	C12	31
29	D8	32
30	D10-1	33
31	D10-2	34
32	D10-3	35

1. A2

```
Human           ATGAATTACGAATTTGAGCGAGAGATTGGTTTTATCAATAGCCAGCCGTCGCTCGCTGAG
Chimp           ATGAATTACGAATTTGAGCGAGAGATTGGTTTTATCAATAGCCAGCCGTCGCTCGCTGAG
Macaque         ATGAATTACGAATTTGAGCGAGAGATTGGTTTTATCAATAGCCAGCCGTCGCTCGCTGAG
Dog             ATGAATTACGAATTTGAGCGAGAGATTGGTTTTATCAATAGCCAGCCGTCGCTCGCTGAG
Cow             ATGAATTACGAATTTGAGCGAGAGATTGGTTTTATCAATAGCCAGCCGTCGCTCGCTGAG
Mouse           ATGAATTACGAATTTGAGCGAGAGATTGGTTTTATCAATAGCCAGCCGTCGCTCGCTGAG
Rat             ATGAATTACGAATTTGAGCGAGAGATTGGTTTTATCAATAGCCAGCCGTCGCTCGCTGAG
Opossum         ATGAATTACGAATTTGAGCGAGAGACTGGCTTTATCAATAGTCAGCCGTCGCTCGCTGAG
Platypus        ATGAATTACGAATTTGAGCGAGAGATTGGTTTTATCAATAGTCAGCCGTCGCTTGCTGAG
Chick           ATGAATTTCGAATTCGAGCGAGAGATCGGTTTTATCAATAGTCAGCCATCGCTTGCTGAG
                ******* ****** **********  ** *********** ***** ***** ******

Human           TGCCTGACATCTTTTCCCCCTGTCGCTGATACATTTCAAAGTTCATCAATCAAGACCTCG
Chimp           TGCCTGACATCTTTTCCCCCTGTCGCTGATACATTTCAAAGTTCATCAATCAAGACCTCG
Macaque         TGCCTGACATCTTTTCCCCCTGTCGCTGATACATTTCAAAGTTCATCAATCAAGACCTCG
Dog             TGCCTGACATCTTTTCCCCCTGTCGCTGATACATTTCAAAGTTCATCAATCAAGACCTCG
Cow             TGCCTGACATCTTTTCCCCCTGTCGCTGATACATTTCAAAGTTCATCAATCAAGACCTCG
Mouse           TGCCTGACATCTTTTCCCCCTGTCGCTGATACATTTCAAAGTTCATCAATCAAGACCTCG
Rat             TGCCTGACATCTTTTCCCCCTGTCGCTGATACATTTCAAAGTTCATCAATCAAGACCTCG
Opossum         TGCCTGACATCTTTTCCCCCTGTCGGTGATACATTTCAAAGTTCATCAATCAAGAGCTCG
Platypus        TGCCTGACATCTTTTCCCCCTGTCGGTGATACATTTCAAAGTTCATCAATCAAGAACTCG
Chick           TGCCTGACATCTTTTCCCCCTGTCGGTGATACATTTCAAAGTTCATCAATCAAGAACTCG
                ************************* ***************************** ****

Human           ACGCTTTCACACTCGACACTGATTCCTCCTCCTTTTGAGCAGACCATTCCCAGCCTGAAC
Chimp           ACGCTTTCACACTCGACACTGATTCCTCCTCCTTTTGAGCAGACCATTCCCAGCCTGAAC
Macaque         ACGCTTTCACACTCGACACTGATTCCTCCTCCTTTTGAGCAGACCATTCCCAGCCTGAAC
Dog             ACGCTTTCACACTCGACACTGATTCCTCCTCCTTTTGAGCAGACCATTCCCAGCCTGAAC
Cow             ACGCTTTCACACTCGACACTGATTCCTCCTCCTTTTGAGCAGACCATTCCCAGCCTGAAC
Mouse           ACGCTTTCACACTCGACACTGATTCCTCCTCCTTTTGAGCAGACCATTCCCAGCCTGAAC
Rat             ACGCTTTCACACTCGACACTGATTCCTCCTCCTTTTGAGCAGACCATTCCCAGCCTGAAC
Opossum         TCGCTTTCACAGTCGACACTGATTCCTCCTCCTTTTGAGCAGACCATCCCCAGCCTGAAC
Platypus        ACTCTTTCACACTCGACACTGATTCCTCCTCCTTTTGAGCAGACTATCCCCAGCCCGAAC
Chick           ACGCTTTCACACTCGACACTGATTCCTCCTCCTTTCGAGCAGACCATCCCCAGCCTGAAC
                 * ******** *********************** ******** ** ******* ****

Human           CCCGGCAGTCACCCTCGCCACGGCGCT------GGCGGCCGCCCCAAGCCGAGCCCCGCG
Chimp           CCCGGCAGTCACCCTCGCCACGGCGCT------GGCGGCCGCCCCAAGCCGAGCCCCGCG
Macaque         CCCGGCAATCACCCTCGCCACGGCGCT------GGCGGCCGCCCCAAGCCGAGCCCCGCG
Dog             CCGGGCAGTCACCCTCGCCACGGCGCT------GGCGGCCGCCCCAAGCCGAGCCCCGCG
Cow             CCGGGCAGTCACCCTCGCCACGGCGCT------GGCGGCCGCCCCAAGCCGAGCCCCGCG
Mouse           CCGGGCAGTCACCCTCGCCACGGCGCTGGCGTTGGCGGCCGCCCCAAGTCGAGCCCCGCG
Rat             CCGGGCAGTCACCCTCGCCAAAGCGCTGGCGCTGGCGGCCGCCCCAAGTCGAGCCCCGCG
Opossum         CCGGGCAGCCACCCTCGCCACGGCAGCGGC---GGCGGTCGCCCCAAGTCGAGCCCCGAT
Platypus        CCAGGCAGCCACCCTCGCCACTGTAGC------AGCGGTTGTCCCAAGCAGATCCCCAAT
Chick           CCCGGCGGCCACCCTCGCCACAGCGGC------GGCGGCCGCCCCAAGGCGAGCCCCCGC
                ** ***   ***********  *           ****  * ******  ** ****

Human           GGCAGCCGC
Chimp           GGCAGCCGC
Macaque         GGCAGCCGC
Dog             GGCAGCCGC
Cow             GGCAGCCGC
Mouse           GGCAGTCGC
Rat             GGCAGTCGC
Opossum         GGCAGCAGC
Platypus        GGCGGCCGC
Chick           GCCCGCAGC
                * * *  **
```

2. A5-1

```
Human           ATGAGCTCTTATTTTGTAAACTCATTTTGCGGTCGCTATCCAAATGGCCCGGACTACCAG
Chimp           ATGAGCTCTTATTTTGTAAACTCATTTTGCGGTCGCTATCCAAATGGCCCGGACTACCAG
Macaque         ATGAGCTCTTATTTTGTAAACTCATTTTGCGGTCGCTATCCAAATGGCCCGGACTACCAG
Dog             ATGAGCTCTTATTTTGTAAACTCATTTTGCGGTCGCTATCCAAATGGCCCGGACTACCAG
Cow             ATGAGCTCTTATTTTGTAAACTCATTTTGCGGTCGCTATCCAAATGGCCCGGACTACCAG
Mouse           ATGAGCTCTTATTTTGTAAACTCATTTTGCGGTCGCTATCCAAATGGCCCGGACTACCAG
Rat             ATGAGCTCTTATTTTGTAAACTCATTTTGCGGTCGCTATCCAAATGGCCCGGACTACCAG
Opossum         ATGAGCTCTTATTTTGTAAACTCATTTTGCGGTCGCTATCCAAATGGCCCGGACTACCAG
Platypus        ATGAGCTCTTATTTTGTAAACTCATTTTGCGGTCGCTATCCAAATGGCCCGGACTACCAG
Chick           ATGAGCTCTTATTTTGTAAACTCATTTTGCGGTCGCTATCCAAATGGCCCGGACTACCAG
                ************************************************************

Human           TTGCATAATTATGGAGATCATAGTTCCGTGAGCGAGCAATTCAGGGACTCGGCGAGCATG
Chimp           TTGCATAATTATGGAGATCATAGTTCCGTGAGCGAGCAATTCAGGGACTCGGCGAGCATG
Macaque         TTGCATAATTATGGAGATCATAGTTCCGTGAGCGAGCAATTCAGGGACTCGGCGAGCATG
Dog             TTGCATAATTATGGAGATCATAGTTCCGTGAGCGAGCAATTCAGGGACTCGGCGAGCATG
Cow             TTGCATAATTATGGAGATCATAGTTCCGTGAGCGAGCAATTCAGGGACTCGGCGAGCATG
Mouse           TTGCATAATTATGGAGATCATAGTTCCGTGAGCGAACAATTCAGGGACTCGGCGAGCATG
Rat             TTGCATAATTATGGAGATCATAGTTCCGTGAGCGAACAATTCAGGGACTCGGCGAGCATG
Opossum         TTACATAATTATGGAGATCACAGTTCCGTGAGCGAGCAATACAGGGATTCAGCGAGCATG
Platypus        TTACATAATTATGGAGATCATAGTTCCGTGAGCGAGCAATACAGGGATTCAGCGAGCATG
Chick           TTACATAATTATGGAGATCACAGCTCGGTGAGCGAGCAATACAGGGACTCCGCCAGCATG
                ** ***************** ** ** ******** **** ****** ** ** ******

Human           CACTCCGGCAGGTACGGCTACGGCTACAATGGCATGGATCTCAGCGTCGGCCGCTCGGGC
Chimp           CACTCCGGCAGGTACGGCTACGGCTACAATGGCATGGATCTCAGCGTCGGCCGCTCGGGC
Macaque         CACTCCGGCAGGTACGGCTACGGCTACAATGGCATGGATCTCAGCGTCGGCCGCTCGGGC
Dog             CACTCCGGCAGGTACGGCTACGGCTACAATGGCATGGATCTCAGCGTCGGCCGCTCAGGC
Cow             CACTCCGGCAGGTACGGCTACGGCTACAATGGCATGGATCTCAGCGTCGGCCGCTCGGGC
Mouse           CACTCCGGCAGGTACGGCTACGGCTACAATGGCATGGATCTCAGCGTCGGCCGTTCGGGT
Rat             CACTCCGGCAGGTACGGCTACGGCTACAATGGCATGGATCTCAGCGTCGGCCGTTCGGGT
Opossum         CATCCCAGCAGGTACGGCTATGGTTACAATGGCATGGATCTCAGCGTTGGGCGCTCAGCT
Platypus        CATTCCAGCAGGTACGGCTACGGCTACAATGGCATGGATCTCAGCGTTGGGCGCTCAGCT
Chick           CACTCCGGCAGGTACGGATACGGCTACAATGGCATGGACCTCAGCGTCGGGCGCTCCGCT
                **  ** ********** ** ** ************** ******** ** ** ** *

Human           TCCGGCCACTTTGGCTCC
Chimp           TCCGGCCACTTTGGCTCC
Macaque         TCCGGCCACTTTGGCTCC
Dog             TCCGGCCACTTTGGCTCC
Cow             TCCGGCCACTTTGGCTCC
Mouse           TCCGGCCACTTTGGCTCC
Rat             TCCGGCCACTTTGGCTCC
Opossum         TCCAGCCACTTTGGTGCC
Platypus        TCCAGCCACTTTGGTGCC
Chick           TCCACCCACTTTGGTGCC
                ***  *********  **
```

3. A5-2

```
Human           GTGGCCCCCTCGCCCGGCAGCGACAGCCACCACGGCGGGAAAAACTCCCTAAGCAACTCC
Chimp           GTGGCCCCCTCGCCCGGCAGCGACAGCCACCACGGCGGGAAAAACTCCCTGAGCAACTCC
Macaque         GTGGCCCCCTCGCCCGGCAGCGACAGCCACCACGGCGGGAAAAACTCCCTGAGCAACTCC
Dog             GTGGCCCCCTCGCCCGGCAGCGACAGCCACCACGGCGGGAAAAACTCCCTGGGCAACTCC
Cow             GTGGCCCCTTCGCCCGGCAGCGACAGCCACCACGGCGGGAAAAACTCCCTGGGCAACTCC
Mouse           GTGGCCCCCTCGCCCGGCAGCGACAGCCACCACGGCGGGAAAAACTCCCTGGGCAACTCC
Rat             GTGGCCCCCTCGCCCGGCAGCGACAGCCACCACGGCGGGAAAAACTCCCTGGGCAACTCC
Opossum         GTGGCCGCTTCGCCTGTCAGCGAGAGTCATCACGGGGTGAAAAACTCGCTAGCTAACTCC
Platypus        GTGGCCACTTCGCCTGTCAGCGAGAGTCATCACGGGGTGAAAAACTCCATAGCTAACTCC
Chick           GTGGCCGCTTCGCCCGTCAGCGAGAGTCACCACGGGGTGAAAAACTCCCTGGGCAACTCC
                ****** * ***** * ****** ** ** ***** * *********  *    ******

Human           AGCGGCGCCTCGGCCGACGCCGGCAGCACCCACATCAGCAGCAGAGAGGGGGTTGGCACG
Chimp           AGCGGCGCCTCGGCCAACGCCGGCAGCACCCACATCAGCAGCAGAGAGGGGGTTGGCACG
Macaque         AGCGGCGCCTCGGCCAACGCCGGCAGCACCCACATCAGCAGCAGAGAGGGGGTTGGCACG
Dog             AGCGGCGCCTCGGCCAACGCCGGCAGCACCCACATCAGCAGCAGAGAGGGGGTTGGCACG
Cow             AGCGGCGCCTCGGCCAACGCCGGCAGCACCCACATCAGCAGCAGAGAGGGGGTTGGCACG
Mouse           AGCGGCGCCTCGGCCAACGCCGGCAGCACCCACATCAGCAGCAGAGAGGGGGTTGGCACG
Rat             AGCGGCGCCTCGGCCAACGCCGGCAGCACCCACATCAGCAGCAGAGAGGGGGTTGGCACG
Opossum         ACGAGCACTTCGTCCAATTCCAGCAACACTCACATA---AGCAGAGAGGGGGTTGGCACC
Platypus        ACTAGCACTTCGTCCAATTCCAGCAGCACTCACATA---AGCAGAGAGGGAGTTGGCACA
Chick           AGCAGCACTTCGTCCAATTCCAGCAGCAGCACGCACATCAGCAGGGACGGGGTTGGCACC
                *   ** * *** ** *  ** *** **           ***** ** ** ********

Human           GCGTCCGGAGCCGAGGAGGACGCC
Chimp           GCGTCCGGAGCCGAGGAGGACGCC
Macaque         GCGTCCGGAGCCGAGGAGGACGCC
Dog             GCGTCCGGAGCCGAGGAGGACGCC
Cow             GCGTCCGGAGCCGAGGAGGACGCC
Mouse           GCGTCCGCAGCCGAGGAGGACGCC
Rat             GCGTCCGCAGCGGAGGAGGACGCC
Opossum         TCGTCTGGGACCGAGGATGACACC
Platypus        TCGTCTGGGACTGAAGATGACACC
Chick           TCGTCCGGCACCGAGGAAGACACT
                 **** *   * ** ** *** *
```

4. A6

```
Human       CTCCCGGACAAGACGTACACCTCACCTTGTTTCTACCAACAGTCCAACTCGGTCCTGGCC
Chimp       CTCCCGGACAAGACGTACACCTCACCTTGTTTCTACCAACAGTCCAACTCGGTCCTGGCC
Macaque     CTCCCGGACAAGACGTACACCTCACCTTGTTTCTACCAACAGTCCAACTCTGTCCTGGCC
Dog         CTCCCGGACAAGACGTACACCTCACCTTGTTTCTACCAACAGTCCAACTCGGTCCTGGCC
Cow         CTCCCGGACAAGACGTACACCTCACCTTGTTTCTACCAACAGTCCAACTCGGTCCTGGCC
Mouse       CTTCCGGACAAGACATACACCTCACCTTGTTTTTACCAACAGTCCAACTCGGTCTTGGCC
Rat         CTTCCGGACAAGACATACACCTCACCTTGTTTCTACCAACAGTCCAACTCGGTCCTGGCC
Opossum     CTCCAGGACAAGACATACACCTCACCTTGTTTCTACCAACAGTCCAACACGGTCATTGCT
Platypus    CTCCAGGACAAGACGTACACCTCACCTTGTTTTTACCAACAGTCCAACACGGTCATTGCC
Chick       CTGCAGGACAAGACCTACACCTCACCTTGTTTCTATCAACAGTCCAACACCGTCATTGCT
            ** * ********* ***************** ** ************ * *** * **

Human       TGCAACCGGGCGTCCTACGAGTACGGGGCCTCGTGTTTCTATTCTGATAAGGACCTCAGT
Chimp       TGCAACCGGGCGTCCTACGAGTACGGGGCCTCGTGTTTCTATTCTGATAAGGACCTCAGT
Macaque     TGCAACCGGGCGTCCTACGAGTACGGGGCCTCGTGTTTCTATTCTGATAAGGACCTCAGT
Dog         TGCAACCGGGCGTCCTACGAGTACGGGGCCTCGTGTTTCTATTCTGATAAGGACCTCAGT
Cow         TGCAACCGGGCATCCTACGAGTACGGGGCCTCATGTTTCTATTCTGATAAGGACCTCAGT
Mouse       TGCAACCGGGCATCCTACGAGTACGGGGCCTCATGTTTCTATTCTGATAAAGACCTCAGT
Rat         TGCAACCGGGCATCCTACGAGTACGGGGCCTCATGTTTCTATTCTGATAAGGACCTCAGT
Opossum     TGCAATCGAGCGTCCTATGAGTACGGCACCTCCTGTTTCTATTCAGAGAAGGACCTGAGT
Platypus    TGCAATCGGGCATCTTATGAGTACGGAGCCTCCTGTTTCTATTCGGAGAAGGACCTAAGT
Chick       TGCAATCGGGCTTCCTATGAGTACGGAGCCTCCTGTTTCTATTCAGACAAGGAAATTAGT
            ***** ** ** ** ** ********  **** *********** ** ** **  * ***

Human       GGCGCCTCGCCCTCGGGCAGTGGCAAGCAGAGGGGCCCCGGGGACTACCTGCACTTTTCT
Chimp       GGCGCCTCGCCCTCGGGCAGTGGCAAGCAGAGGGGCCCCGGGGACTACCTGCACTTTTCT
Macaque     GGCGCCTCGCCCTCGGGCAGTGGCAAGCAGAGGGGCCCCGGGGACTACCTGCACTTTTCT
Dog         GGCGCCTCGCCCTCGGGCAGTGGCAAGCAGAGGGGCCCCGGGGACTACCTGCACTTTTCT
Cow         GGCGCCTCGCCCTCGGGCAGTGGCAAACAGAGGGGCCCCGGGGACTACCTGCACTTTTCT
Mouse       GGCGCCTCACCCTCGGGCAATAACAAGCAGAGGGGCCCCGGGGACTACCTGCACTTTTCT
Rat         GGCGCCTCACCCTCGGGCAATAGCAAGCAGAGGGGCCCCGGGGACTACCTGCACTTTTCT
Opossum     AGCGCTTCTCCCTCGGGCAGTGGCAAACAGAGGGGCCACGGGGAGTATCTGCACTTTTCT
Platypus    AACGCCTCTCCCTCCGGCAGTGGCAAGCAGAGAGGCCACGGGGATTACCTGCACTTTTCT
Chick       AGCGCCTCTCCCTCCGGCAGTGGCAAGCAGAGGGGACAAGGGGACTATCTGCACTTTTCT
              *** ** ***** **** *  *** ***** ** *  ***** ** ************

Human       CCCGAGCAGCAGTACAAACCCGACAGCAGCAGCGGGCAGGGCAAA
Chimp       CCCGAGCAGCAGTACAAACCCGACAGCAGCAGCGGGCAGGGCAAA
Macaque     CCCGAGCAGCAGTACAAACCCGACAGCAGCAGCGGGCAGGGCAAA
Dog         CCCGAGCAGCAGTACAAACCCGACAGCAGCAGCGTGCCGGGCAAA
Cow         CCCGAGCAGCAGTACAAACCCGACAGCAGCAGCGTGCCGGGCAAA
Mouse       CCCGAGCAGCAGTACAAACCTGACGGC---AGCGTGCAGGGCAAA
Rat         CCCGAGCAGCAGTACAAACCTGACAGCAGCAGCGTGCAGGGCAAA
Opossum     CCTGAGCAACAATACAAATCCGAAAGC---AGCGTGCAGGGCAAA
Platypus    CCAGAACAACAATACAAATCCGACAGC---AGCGTGCAGAGCAAA
Chick       CCCGAGCAACAATACAAATCTAAC------GGCGTGCAAAGCAAA
            ** ** ** ** ****** *  *        *** **   *****
```

5. A9

```
Human           GCGCTCTCCTTCGCGGGCTTGCCCTCCAGCCGGCCTTATGGCATTAAACCTGAACCGCTG
Chimp           GCGCTCTCCTTCGCGGGCTTGCCCTCCAGCCGGCCTTATGGCATTAAACCTGAACCGCTG
Macaque         GCGCTCTCCTTCGCGGGCTTGCCCTCCAGCCGGCCTTATGGCATTAAACCTGAACCACTG
Dog             GCGCTCTCCTTCGCGGGCTTGCCCTCCAGCCGGCCTTACGGCATTAAACCTGAACCACTG
Cow             GCGCTCTCCTTCGCGGGCTTGCCCTCCAGCCGGCCTTATGGCATTAAACCTGAACCGCTG
Mouse           GCGCTCTCCTTCGCGGGCTTACCCTCCAGCCGGCCTTATGGCATTAAACCTGAACCGCTC
Rat             GCGCTCTCCTTCGCGGGCTTACCCTCCAGCCGGCCTTATGGCATTAAACCTGAACCGCTG
Opossum         TCTCTGTCCTTCCCGGGTTTACCTTCCAGCAGGCATTACGGCATTAAACCTGAACCGCTC
Platypus        TCCCTGTCTTTCCCGGGGTTGCCTTCGAGCAGGCATTACGGCATTAAACCTGAACCGCTC
Chick           TCCTTGTCCTTCCCCGGGTTACCTACCAGCAGGCATTACGGCATTAAACCTGAACCGCTG
                 *  * ** *** * ** ** **  * *** *** *** ***************** **

Human           TCGGCCAGAAGGGGTGACTGTCCCACGCTTGACACTCACACTTTGTCCCTGACTGACTAT
Chimp           TCGGCCAGAAGGGGTGACTGTCCCACGCTTGACACTCACACTTTGTCCCTGACTGACTAT
Macaque         TCGGCCAGAAGGGGTGACTGTCCCACGCTTGACACTCACACTTTGTCCCTGACTGACTAT
Dog             TCCGCCAGAAGGGGTGACTGTCCCACGCTTGACACTCACACTTTGTCCCTGACTGACTAT
Cow             TCGGCCAGAAGGGGTGACTGTCCCACGCTTGACACTCACACTTTGTCCCTGACTGACTAT
Mouse           TCGGCCAGAAGGGGTGACTGTCCCACGCTTGACACTCACACTTTGTCCCTGACTGACTAT
Rat             TCGGCCAGAAGGGGTGACTGTCCCACGCTTGACACTCACACTTTGTCCCTGACTGACTAT
Opossum         TCGGCCAGAAGGGGTGACTGTACCACGTTTGACACTCACACTTTGTCTCTGTCTGACTAT
Platypus        TCGGCCAGAAGGGGTGACTGTACCACGTTTGACACTCACACTTTGTCTCTGTCTGACTAT
Chick           GCGGCCAGAAGGGGTGACTGTACCACGTTTGACACTCACACTTTGTCTCTGTCTGATTAT
                 * ****************** ***** ******************* *** **** ***

Human           GCTTGTGGTTCTCCTCCAGTTGATAGAGAAAAA
Chimp           GCTTGTGGTTCTCCTCCAGTTGATAGAGAAAAA
Macaque         GCTTGTGGTTCTCCTCCAGTTGATAGAGAAAAA
Dog             GCTTGTGGTTCTCCTCCAGTTGATAGAGAAAAA
Cow             GCTTGTGGTTCTCCTCCAGTTGATAGAGAAAAA
Mouse           GCTTGTGGTTCTCCTCCAGTTGATAGAGAAAAA
Rat             GCTTGTGGTTCTCCTCCAGTTGATAGAGAAAAA
Opossum         GCTTGTGGTTCTCCTCCCAGATGATAGAGGATA
Platypus        GCTTGTGGTTCTCCTCCAGTTGATAGGGATAAG
Chick           GCTTGTGGTTCTCCTCCAGTTGATAGGGATAAG
                *****************             *
```

6. A11

```
Human           CCCCAGACCCCGTCTTCGCGCCCAATGACATACTCCTACTCCTCCAACCTGCCCCAGGTC
Chimp           CCCCAGACCCCGTCTTCGCGCCCAATGACATACTCCTACTCCTCCAACCTGCCCCAGGTC
Macaque         CCCCAGACCCCGTCTTCGCGCCCAATGACATACTCCTACTCCTCCAACCTGCCCCAGGTC
Dog             CCCCAGACCCCGTCTTCGCGCCCAATGACATACTCCTACTCTTCCAACCTGCCCCAGGTC
Cow             CCCCAGACCCCGTCTTCGCGCCCAATGACATACTCCTACTCTTCCAACCTGCCCCAGGTC
Mouse           CCCCAGACCCCGTCTTCGCGCCCAATGACATACTCCTACTCCTCCAACCTGCCCCAGGTC
Rat             CCCCAGACCCCGTCTTCGCGCCCAATGACATACTCCTACTCCTCCAACCTGCCCCAGGTC
Opossum         CCCCAAACCCCGTCTTCTCGCCCCATGACATACTCCTACTCGTCCAACCTCCCCCAGGTC
Platypus        CCCCAGACCCCGTCTTCTCGCCCTATGACATATTCCTACTCCTCCAACCTGCCCCAGGTC
Chick           CCCCAGACCCCGTCTTCTCGCCCTATGACATACTCCTACTCCTCCAACCTGCCCCAGGTC
                ***** *********** ***** ******** ******** ******** *********

Human           CAACCCGTGCGCGAAGTGACCTTCAGAGAGTACGCCATTGAGCCCGCCACTAAATGGCAC
Chimp           CAACCCGTGCGCGAAGTGACCTTCAGAGAGTACGCCATTGAGCCCGCCACTAAATGGCAC
Macaque         CAACCCGTGCGCGAAGTGACCTTCAGAGAGTACGCCATTGAGCCCGCCACTAAATGGCAC
Dog             CAACCCGTACGCGAAGTGACCTTCAGGGAGTACGCCATTGAGCCCGCCACTAAATGGCAC
Cow             CAACCCGTGCGCGAAGTGACCTTCAGAGAATACGCCATTGAGCCCGCCACTAAATGGCAC
Mouse           CAACCCGTGCGCGAAGTGACCTTCAGAGAGTACGCCATTGAGCCCGCCACTAAATGGCAC
Rat             CAACCCGTGCGCGAAGTGACCTTCAGAGAATACGCCATTGAGCCCGCCACTAAATGGCAC
Opossum         CAACCTGTAAGAGAAGTAACCTTCCGGGAGTACGCCATTGAGCCCTCCAGTAAATGGCAC
Platypus        CAACCTGTGAGAGAAGTTACCTTCAGGGAGTACGCCATTGATCCCTCCAGTAAATGGCAT
Chick           CAACCCGTGAGAGAAGTGACCTTCAGGGAATATGCCATTGATCCCTCCAGTAAATGGCAC
                ***** **  * ***** ****** * ** ** ******** *** *** *********

Human           CCCCGCGGCAATCTGGCCCACTGCTACTCCGCGGAGGAGCTCGTGCACAGAGACTGCCTG
Chimp           CCCCGCGGCAATCTGGCCCACTGCTACTCCGCGGAGGAACTCGTGCACAGAGACTGCCTG
Macaque         CCCCGCGGCAATCTGGCCCACTGCTACTCCGCGGAGGAGCTCGTGCACAGAGACTGCCTG
Dog             CCCCGCGGCAATCTGGCTCACTGCTACTCCGCGGAGGAGCTCGTGCACAGAGACTGCCTG
Cow             CCCCGCGGCAATCTGGCCCACTGCTACTCCGCGGAGGAGCTCGTGCACAGAGACTGCCTG
Mouse           CCCCGCGGCAATCTGGCCCACTGCTACTCCGCGGAGGAGCTCGTGCACAGAGACTGTCTG
Rat             CCCCGCGGCAATCTGGCCCACTGCTACTCCGCGGAGGAGCTCGTGCACAGAGACTGTCTG
Opossum         CCCCGGGCCAACCTGCCCCACTGCTATTCCGCAGAGGAGCTCATGCACAGAGACTGCCTG
Platypus        CCCCGGAGCAATCTGCCCCACTGCTACTCAGCGGAGGAGATCATGCACAGAGACTGCCTG
Chick           CCCCGGAACAACCTGCCCCACTGCTACTCCGCAGAGGAGATCATGCACAGAGACTGCTTG
                *****   *** *** * ******** ** ** *****  ** *************  **

Human           CAGGCGCCCAGCGCGGCCGGCGTGCCTGGCGACGTGCTGGCCAAGAGCTCGGCCAACGTC
Chimp           CAGGCGCCCAGCGCGGCCGGCGTGCCTGGCGACGTGCTGGCCAAGAGCTCGGCCAACGTC
Macaque         CAGGCGCCCAGCGCGGCCGGCGTGCCTGGCGACGTGCTGGCCAAGAGCTCCGCCAACGTC
Dog             CAGGCGCCCAGCGCGGCCGGCGTGCCTGGCGACGTGCTGGCCAAGAGCTCGGCCAACGTC
Cow             CAGGCGCCCAGCGCGGCCGGCGTGCCTGGCGACGTTCTGGCCAAGAGCTCGGCCAACGTC
Mouse           CAGGCGCCCAGCGCGGCCGGCGTGCCTGGCGACGTGCTGGCCAAGAGCTCGGCCAACGTC
Rat             CAGGCGCCCAGCGCGGCCGGCGTGCCTGGCGACGTGCTGGCCAAGAGCTCGGCCAACGTC
Opossum         CCCGCC---TCCGGCAGCGCGAGCGTGGGCGACATGCTGGCCAAGAACTCCGCCAACGTC
Platypus        CCGGCT---ACCAACACGGCCAGCGTGGGCGACATGCTCGCCAAGAACTCGGCCAACGTC
Chick           CCTTCC---ACCACCACCGCCAGCATGGGCGAGGTGTTCGGCAAAAGCACCGCGAACGTC
                *   *      *      *        *****  *  * * *** * * * ** ******

Human           TACCACCACCCCACCCCC
Chimp           TACCACCACCCCACCCCC
Macaque         TACCACCACCCCACCCCC
Dog             TACCACCACCCCACCCCC
Cow             TACCACCACCCCACCCCC
Mouse           TACCACCACCCCACCCCC
Rat             TACCACCACCCCACCCCC
Opossum         TACCACCACCCCAGCACC
Platypus        TACCACCACCCCACCTCC
Chick           TACCACCACCCCAGCGCC
                ************* * **
```

7. A13-1

```
Human           ATGACAGCCTCCGTGCTCCTCCACCCCCGCTGGATCGAGCCCACCGTCATGTTTCTCTAC
Chimp           ATGACAGCCTCCGTGCTCCTCCACCCCCGCTGGATCGAGCCCACCGTCATGTTTCTCTAC
Macaque         ------------------------------------------------------------
Dog             ATGACAGCCTCCGTGCTCCTCCACCCCCGCTGGATCGAGCCCACCGTCATGTTTCTCTAC
Cow             ATGACAGCCTCCGTGCTCCTCCACCCCCGCTGGATCGAGCCCACCGTCATGTTTCTCTAC
Mouse           ATGACAGCCTCCGTGCTCCTCCACCCCCGCTGGATCGAGCCCACCGTCATGTTTCTCTAC
Rat             ATGACAGCCTCCGTGCTCCTCCACCCCCGCTGGATCGAGCCCACCGTCATGTTTCTCTAC
Opossum         ATGACAGCCTCCGTGCTCCTCCACCCCCGCTGGATCGAGCCCACCGTCATGTTCCTCTAC
Platypus        ------------------------------------------------------------
Chick           ------------------------------------------------ATGTTCCTCTAC


Human           GACAACGGCGGCGGCCTGGTGGCCGACGAGCTCAACAAGAACATGGAAGGGGCGGCGGCG
Chimp           GACAACGGCGGCGGCCTGGTAGCCGACGAGCTCAACAAGAACATGGAAGGGGCGGCGGCG
Macaque         ------------------------------------------------------------
Dog             GACAACGGCGGTGGCTTGGTGGCCGACGAACTCAACAAGAACATGGAAGGGGCGGCGGCG
Cow             GACAACGGCGGCGGCCTGGTGGCCGACGAGCTCAACAAGAACATGGAAGGGGCGGAGAAG
Mouse           GACAACGGCGGCGGCCTGGTGGCCGACGAGCTCAACAAGAACATGGAAGGGGCGGCGGCG
Rat             GACAACGGCGGCGGCCTGGTGGCCGACGAGCTCAACAAGAACATGGAAGGGGCGGCGGCG
Opossum         GACAACGGCGGCGGCCTGGTGGCGGACGAGCTCAACAAAAACATGGAAGGGGCGGCGGCC
Platypus        ------------------------------------------------------------
Chick           GACAACAGC------------CTGGATGAGATCAATAAGAACATGGAC------------
```

8. A13-2

```
Human           GGTCTTCCCATGGAAAGCTACCAGCCCTGGGCGCTGCCCAACGGCTGGAACGGCCAAATG
Chimp           GGTCTTCCCATGGAAAGCTACCAGCCCTGGGCGCTGCCCAACGGCTGGAACGGCCAAATG
Macaque         ------------------------------------------------------------
Dog             GGTCTTCCCATGGAAAGCTACCAGCCCTGGGCGCTGCCCAACGGCTGGAACGGCCAAATG
Cow             GGTCTTCCCATGGAAAGCTACCAGCCCTGGGCGCTGCCCAACGGCTGGAACGGCCAAATG
Mouse           GGGCTTCCCATGGAAAGCTATCAGCCCTGGGCTCTGCCCAACGGCTGGAACGGCCAAATG
Rat             GGGCTTCCTATGGAAAGCTATCAGCCCTGGGCTCTGCCCAACGGCTGGAACGGCCAAATG
Opossum         GGGCTGCCCATGGAAAGCTACCAGCCCTGGGCTTTGCCCAACGGCTGGAATGGCCAGGTG
Platypus        GGGCTGCCCATGGAGAGCTACCAGCCCTGGGCGCTGCCCAACGGCTGGAACGGCCAGGTG
Chick           ---CTCCCCATGGACAGCTACCAGCCGTGGGCCATCACCAACGGGTGGAACGGGCAAGTG

Human           TACTGCCCCAAAGAGCAGGCGCAGCCTCCCCACCTCTGGAAGTCCACTCTGCCCGACGTG
Chimp           TACTGCCCCAAAGAGCAGGCGCAGCCTCCCCACCTCTGGAAGTCCACTCTGCCCGACGTG
Macaque         ------------------------------------------------------------
Dog             TACTGCCCCAAAGAGCAGGCGCAGCCTCCCCACCTCTGGAAGTCCACTCTGCCCGACGTG
Cow             TACTGCCCCAAAGAGCAGGCGCAGCCTCCCCACCTCTGGAAGTCCACTCTGCCCGACGTG
Mouse           TACTGCCCCAAAGAGCAGACGCAGCCTCCCCACCTCTGGAAGTCCACTCTGCCCGACGTC
Rat             TACTGCCCCAAAGAGCAGACGCAGCCTCCCCACCTCTGGAAGTCCACTCTGCCCGACGTC
Opossum         TACTGCCCCAAAGAGCAAGGCCAGCCTCCCCACCTCTGGAAGTCCACTCTACCGGATGTT
Platypus        TACTGCCCCAAAGAGCAGCCCCAGCCTCCCCACCTCTGGAAGTCCACCCTGCCCGACGTG
Chick           TACTGCCCCAAGGAGCAGAGCCAGCCGCCTCACCTCTGGAAGTCCACCCTCCCGGACGTC

Human           GTC
Chimp           GTC
Macaque         ---
Dog             GTC
Cow             GTC
Mouse           GTC
Rat             GTC
Opossum         GTC
Platypus        GTT
Chick           GTC
```

9. B2

```
Human          ATGAATTTTGAATTTGAGAGGGAGATTGGGTTTATAAACAGCCAGCCGTCGCTCGCCGAG
Chimp          ATGAATTTTGAATTTGAGAGGGAGATTGGGTTTATAAACAGCCAGCCGTCGCTCGCCGAG
Macaque        ATGAATTTTGAATTTGAGAGGGAGATTGGGTTTATAAACAGCCAGCCATCGCTCGCCGAG
Dog            ATGAATTTTGAATTTGAGAGGGAGATTGGGTTTATAAACAGCCAGCCATCGCTCGCCGAG
Cow            ATGAATTTTGAATTTGAGAGGGAGATTGGGTTTATAAACAGCCAGCCATCGCTCGCCGAG
Mouse          ATGAATTTTGAATTTGAGAGGGAGATTGGGTTTATAAACAGCCAGCCATCGCTCGCCGAG
Rat            ATGAATTTTGAATTTGAGAGGGAGATTGGGTTTATAAACAGCCAGCCATCGCTCGCCGAG
Opossum        ATGAATTTTGAATTTGAGAGGGAGATTGGGTTTATAAACAGCCAGCCATCGCTCGCGGAG
Platypus       ATGAATTTTGAATTCGAGAGGGAGATTGGGTTTATCAACAGTCAGCCTTCGCTCGCCGAG
Chick          ------------------------------------------------------------

Human          TGTCTGACTTCCTTCCCCGCTGTCTTGGAGACATTTCAAACTTCATCAATCAAGGAGTCG
Chimp          TGTCTGACTTCCTTCCCCGCTGTCTTGGAGACATTTCAAACTTCATCAATCAAGGAGTCG
Macaque        TGTCTGACTTCCTTCCCCGCTGTCTTGGAGACATTTCAAACTTCATCAATCAAGGAGTCG
Dog            TGTCTGACTTCCTTCCCCGCTGTCTTGGAGACATTTCAAACTTCATCAATCAAGGAGTCG
Cow            TGTCTGACTTCCTTCCCCGCTGTCTTGGAGACATTTCAAACTTCATCAATCAAGGAGTCG
Mouse          TGTCTGACTTCCTTCCCCGCTGTCTTGGAGACATTTCAAACTTCATCAATCAAGGAGTCG
Rat            TGTCTGACTTCCTTCCCCGCTGTCTTGGAGACATTTCAAACTTCATCAATCAAGGAGTCG
Opossum        TGCCTGACTTCCTTCCCCGCTGTCTTGGAGACATTTCAAACTTCATCAATCAAGGAGTCG
Platypus       TGCCTGACTTCCTTCCCCGCTGTCCTGGAAACATTTCAAACTTCATCAATCAAGGAGTCG
Chick          ------------------------------------------------------------

Human          ACATTAATTCCTCCT---CCTCCTCCTTTCGAGCAAACCTTCCCCAGCCTCCAGCCCGGC
Chimp          ACATTAATTCCTCCT---CCTCCTCCTTTCGAGCAAACCTTCCCCAGCCTCCAGCCCGGC
Macaque        ACATTAATTCCTCCT---CCTCCTCCTTTCGAGCAAACCTTCCCCAGCCTCCAGCCCGGC
Dog            ACATTAATTCCTCCT---CCTCCTCCTTTCGAGCAAACCTTCCCCAGCCTCCAGCCCGGC
Cow            ACATTAATTCCTCCT---CCTCCTCCTTTCGAGCAAACCTTCCCCAGCCTCCAGCCCGGC
Mouse          ACATTAATTCCTCCT---CCTCCTCCTCTGGAGCAAACCTTCCCCAGCCTCCAGCTCGGC
Rat            ACATTAATTCCTCCTCCTCCTCCTCCTCTGGAGCAAACCTTCCCCAGCCTCCAGCTCGGC
Opossum        ACATTAATTCCTCCT---CCTCCTCCTTTCGAGCAAACCATCCCCAGCCTCCAGCCAGGC
Platypus       TCGCTAATTCCTCTC---------CCCTTTGAGCAGAGC---------------------
Chick          ------------------------------------------------------------

Human          GCCTCCACCCTTCAGAGACCCAGGAGCCAAAAGCGAGCCGAAGATGGG
Chimp          GCCTCCACCCTTCAGAGACCCAGGAGCCAAAAGCGAGCCGAAGATGGA
Macaque        GCCTCCACCCTTCAGAGACCCAGGAGCCAAAAGCGAGCCGAAGATGGG
Dog            ACCTCCACCCTTCAGAGACCCGGGAGCCAAAAGCGAGCCGAAGATGGG
Cow            GCCTCCACCCTTCAGAGACCCGGGAGCCAAAAGCGAGCCGAAGATGGG
Mouse          GCCTCCACCCTTCAGAGACCAGGGAGCCAAAAGCAAGCCGGAGATGGG
Rat            GCCTCCACCCTTCAGAGACCAGGGAGCCAAAAGCCAGCCGGAGATGGG
Opossum        GCCTCCACCCTTCAGAGACCCCGGAGCCAAAAGAGAACCGATGATGGG
Platypus       ------------------------------------------------
Chick          ------------------------------------------------
```

10. B4

```
Human          GAGGCGGTCAGCAGCAGCCCCCCGCCGCCTCCCTGCGCCCAGAACCCCCTGCACCCCAGC
Chimp          GAGGCGGTCAGCAGCAGCCCCCCGCCGCCTCCCTGCGCCCAGAACCCCCTGCACCCCAGC
Macaque        GAGGCGGTCAGCAGCAGCCCCCCGCCGCCTCCCTGCGCCCAGAACCCCCTGCACCCCAGT
Dog            GAGGCGGTCAGCAGCAGCCCCCCGCCGCCTCCCTGCGCCCAGAACCCCCTGCACCCCAGC
Cow            GAGGCGATCAGCAGCAGCCCCCCGCCGCCTCCCTGCGCCCAGAACCCCCTGCACCCCAGC
Mouse          GAGGCGGTCAGCAGCAGCCCCCCGCCGCCTCCCTGCGCCCAGAACCCCCTGCATCCCAGC
Rat            GAGGTGGTCAGCAGCAGTCCCCCGCCGCCTCCCTGCGCCCAGAACCCCCTGCATCCCAGC
Opossum        GAGGCGGTCAGCCCCAGCCCCCCGCCACCGCCCTGTGGTCAAAACCCTCTACACCCCAGC
Platypus       GAGGCGGTCAGCCCGAGCCCCCCGCCTCCACCTTGTAGCCAAAACGCCCTGCACCCCAGC
Chick          GAGCCGGGCACCCCCAGC---CCTCCACCCTCCTGCAGCCAAAACTCTCTGAACCAAAGC
               ***  *  ** *   **    ** ** **  * **    ** *** * **  * *  **

Human          CCGTCCCACTCCGCGTGCAAAGAGCCCGTCGTCTACCCCTGGATGCGCAAAGTTCACGTG
Chimp          CCGTCCCACTCCGCGTGCAAAGAGCCCGTCGTCTACCCCTGGATGCGCAAAGTTCACGTG
Macaque        CCGTCCCACTCCGCGTGCAAAGAGCCAGTCGTCTACCCCTGGATGCGCAAAGTTCACGTG
Dog            CCTTCCCACTCCGCGTGCAAAGAGCCCGTCGTCTACCCCTGGATGCGCAAAGTTCACGTG
Cow            CCGTCCCACTCCGCGTGCAAAGAGCCCGTCGTCTACCCCTGGATGCGCAAAGTTCACGTG
Mouse          CCGTCCCACTCCGCGTGCAAAGAGCCCGTCGTCTACCCCTGGATGCGCAAAGTTCACGTG
Rat            CCGTCCCACTCCGCGTGCAAAGAGCCCGTCGTCTACCCCTGGATGCGCAAAGTTCACGTG
Opossum        CCATCCCACTCCTCTTGCAAAGAACCCGTAGTCTATCCCTGGATGCGGAAAGTTCACGTG
Platypus       CCTTCCCACTCCTCTTGCAAAGAGCCCATAGTCTACCCATGGATGAGAAAGGTTCATGTG
Chick          CCTTCCAATTCCTCTTGCAAAGAGCCGGTAGTTTACCCCTGGATGAAAAAAGTCCATGTA
               ** *** * *** * ******** **  * ** ** ** ******   ** ** ** **

Human          AGCACGGTAAACCCCAATTACGCCGGCGGGGAGCCCAAGCGCTCTCGGACCGCCTAC
Chimp          AGCACGGTAAACCCCAATTACGCCGGCGGGGAGCCCAAGCGCTCTCGGACCGCCTAC
Macaque        AGCACGGTAAACCCCAATTACGCCGGCGGGGAGCCCAAGCGCTCTCGGACCGCCTAC
Dog            AGCACGGTAAACCCCAATTACGCCGGCGGGGAGCCCAAGCGCTCTCGGACAGCCTAC
Cow            AGCACGGTCAACCCCAATTACGCCGGCGGGGAGCCCAAGCGCTCTCGGACCGCCTAC
Mouse          AGCACGGTAAACCCCAATTACGCCGGCGGGGAGCCCAAGCGCTCTCGGACCGCCTAC
Rat            AGCACGGTAAACCCCAATTACGCCGGCGGGGAGCCCAAGCGCTCCCGGACCGCCTAC
Opossum        AGCACCGTAAATCCCAATTACAACGGAGGGGAGCCAAAGCGCTCCCGGACAGCTTAT
Platypus       AGCACGGTAAACCCCAATTACACGGGAGGGGAACCGAAGCGCTCTCGGACCGCTTAC
Chick          AGCACGGTAAACCCCAATTATTCAGGAGGGGAACCGAAACGCTCGCGCACAGCCTAC
               ***** ** ** ********    ** ***** ** ** ***** ** ** ** **
```

11. B5

```
Human           ATGAGCTCGTACTTTGTAAACTCCTTCTCGGGGCGTTATCCAAATGGCCCGGACTATCAG
Chimp           ATGAGCTCGTACTTTGTAAACTCCTTCTCGGGGCGTTATCCAAATGGCCCGGACTATCAG
Macaque         ATGAGCTCGTACTTTGTAAACTCCTTCTCGGGGCGTTATCCAAATGGCCCGGACTATCAG
Dog             ATGAGCTCGTACTTTGTAAACTCCTTCTCGGGGCGTTATCCAAATGGCCCGGACTATCAG
Cow             ATGAGCTCGTACTTTGTAAACTCCTTCTCGGGGCGTTATCCAAATGGCCCGGACTATCAG
Mouse           ATGAGCTCGTACTTTGTAAACTCCTTCTCGGGGCGTTATCCAAATGGCCCGGACTATCAG
Rat             ATGAGCTCGTACTTTGTAAACTCCTTCTCGGGGCGTTATCCAAATGGCCCGGACTATCAG
Opossum         ATGAGCTCTTACTTTGTAAACTCGTTCTCGGGGCGCTATCCAAATGGCCCGGACTATCAG
Platypus        ATGAGCTCTTACTTTGTAAACTCGTTCTCGGGGCGCTATCCAAATGGCCCGGAATATCAG
Chick           ATGAGCTCTTACTTTGTAAACTCGTTCTCAGGGCGCTACCCAAATGGCCCCGACTATCAG
                ******** ************** ***** ***** ** *********** ** ******

Human           TTGCTAAATTATGGCAGTGGCAGCTCTCTGAGCGGCTCTTACAGGGATCCCGCTGCCATG
Chimp           TTGCTAAATTATGGCAGTGGCAGCTCTCTGAGCGGCTCTTACAGGGATCCCGCTGCCATG
Macaque         TTGCTAAATTATGGCAGTGGCAGCTCTCTGAGCGGCTCTTACAGGGATCCCGCTGCCATG
Dog             TTGCTAAATTATGGCAGTGGCAGCTCTCTGAGCGGCTCTTACAGGGATCCCGCTGCCATG
Cow             TTGCTAAATTATGGCAGTGGCAGCTCTCTGAGCGGCTCTTACAGGGATCCCGCTGCCATG
Mouse           TTGCTAAATTATGGCAGTGGCAGCTCTCTGAGCGGCTCTTACAGGGATCCCGCTGCCATG
Rat             TTGCTAAATTATGGCAGTGGCAGCTCTCTGAGCGGCTCTTACAGGGATCCCGCTGCCATG
Opossum         TTGCTAAATTATGGCAGTAGCAGCTCTTTGAACGGTTCTTACAGGGATTCTGCTACCATG
Platypus        TTGCTAAATTATGGGGCTGGCAGCTCTCTG-ACGGTTCGTACAGGGATTCAGGCACCATG
Chick           TTACTAAATTATGGGACCAGCAGTTCCATGAACGGTTCTTACAGAGATTCAAGCACCATG
                ** ***********     **** **  **  *** ** ***** *** *     *****

Human           CACACCGGCTCTTACGGCTACAATTACAATGGGATGGACCTCAGCGTCAACCGCTCCTCG
Chimp           CACACCGGCTCTTACGGCTACAATTACAATGGGATGGACCTCAGCGTCAACCGCTCCTCG
Macaque         CACACCGGCTCTTACGGCTACAATTACAATGGGATGGACCTCAGCGTCAACCGCTCCTCG
Dog             CACACCGGCTCTTACGGCTACAATTACAATGGGATGGACCTCAGCGTCAACCGCTCCTCG
Cow             CACACCGGCTCTTACGGCTACAATTACAATGGGATGGACCTCAGCGTCAACCGCTCCTCG
Mouse           CACACCGGCTCTTACGGCTACAATTACAATGGGATGGATCTCAGCGTCAACCGCTCCTCG
Rat             CACACCGGCTCTTACGGCTACAATTACAATGGGATGGATCTCAGCGTCAACCGCTCCTCG
Opossum         CATACCAGCTCTTACGGCTACAATTACAATGGGATGGATCTCAGCATCAACCGCTCCTCA
Platypus        CATACCGGCTCTTATGGCTACAACTACAATGGAATGGATCTTAGCATCAACCGT---TCA
Chick           CATTCCAGCTCTTATGGCTACAACTACAATGGGATGGACCTTAGCATCAACCGC---TCA
                **  ** ******* ******** ******** ***** ** *** *******    **

Human           GCCTCCTCCAGCCACTTTGGGGCGGTGGGCGAGAGCTCGCGCGCCTTCCCCGCGCCCGCC
Chimp           GCCTCCTCCAGCCACTTTGGGGCGGTGGGCGAGAGCTCGCGCGCCTTCCCCGCGCCCGCC
Macaque         GCCTCCTCCAGCCACTTTGGGGCGGTGGGCGAGAGCTCGCGCGCCTTCCCCGCGCCCGCC
Dog             GCCTCCTCCAGCCACTTTGGGGCGGTGGGCGAGAGCTCGCGCGCCTTCCCCGCGCCCGCC
Cow             GCCTCCTCCAGCCACTTTGGGGCGGTGGGCGAGAGCTCGCGCGCCTTCCCTGCGCCCGCC
Mouse           GCCTCCTCCAGCCACTTTGGGGCGGTGGGCGAGAGCTCGCGCGCCTTCCCCGCGTCCGCC
Rat             GCCTCATCCAGCCACTTTGGGGCGGTGGGCGAGAGCTCGCGCGCCTTCCCCGCGTCCGCC
Opossum         GCTTCCTCCAGCCACTTTGGGGCGGTGGGGGAGACCTCGCGTGCCTTCCCCTCGCCGGGC
Platypus        GCCTCCCTCTGCCACTTTGGGGCGGTGGGCGAGACCTCGCGCGGTTTCCCCCGCCCCGCC
Chick           GCCTCCTCTAGTCACTTTGGGGCTGTGGGGGAGAGCTCCCGCGGTTTCCCTTCTCCAGCT
                ** **     * *********** ***** **** *** ** *  *****     * *

Human           CAGGAGCCCCGCTTCAGGCAAGCG
Chimp           CAGGAGCCCCGCTTCAGGCAAGCG
Macaque         CAGGAGCCCCGCTTCAGGCAAGCG
Dog             CAGGAGCCCCGCTTCAGGCAAGCG
Cow             CAGGAGCCCCGCTTCAGGCAAGCG
Mouse           CAGGAACCCCGCTTCAGGCAGGCG
Rat             CAGGAGCCCCGCTTCAGGCAGGCG
Opossum         CAGGAGTCCCGGTTCAGGCAGGCT
Platypus        CAGGAGAACAGGTTTAGACAGACG
Chick           CAGGAGAGCAGGTTTAGACAGGCG
                *****   * * ** ** **  *
```

12. B7-1

```
Human           GAACAAACTTCTTGTGCGTTTGCTTCCAACCCCCAGCGCCCGGGCTATGGAGCGGGTTCG
Chimp           GAACAAACTTCTTGTGCGTTTGCTTCCAACCCCCAGCGCCCGGGCTATGGAGCGGGTTCG
Macaque         GAACAAACTTCTTGTGCGTTTGCTTCCAACCCCCAGCGCCCGGGCTATGGAGCGGGTTCG
Dog             GAACAAACTTCTTGTGCGTTTGCTTCCAACCCCCAGCGCCCGGGCTATGGAGCGGGTTCG
Cow             GAACAAACTTCTTGTGCGTTTGCTTCCAACCCCCAGCGCCCGGGCTATGGAGCGGGTTCG
Mouse           GAACAAACTTCTTGCGCCTTTGCTTCCAACCCCCAGCGCCCGGGCTATGGAGCAGGTCCG
Rat             GAACAAACTTCTTGCGCCTTTGCCTCCAACCCCCAGCGCCCGGGCTATGGAGCAGGTCCG
Opossum         GAACAAACTTCTTGCGCTTTCGCACCCAACGCCCAGCGCCCGGGCTATGGAGCTGGCTCG
Platypus        GAACAAACTTCTTGCGCGTTCGCTTCCAGCGCGCAGCGCCAGGGCTATGGAGCGGGCTCG
Chick           GAGCAAACTTCTTGCGCTTTCGCCTCCAACACCCAACGAGCGGGCTATGGGGCCGGCTCC
                ** *********** ** ** **  *** * * ** **   ********* ** **  *

Human           GGCGCTTCCTTCGCCGCCTCGATGCAGGGCTTGTACCCCGGCGGGGGGGGCATGGCGGGC
Chimp           GGCGCTTCCTTCGCCGCCTCGATGCAGGGCTTGTACCCCGGCGGGGGGGGCATGGCGGGC
Macaque         GGCGCTTCCTTCGCCGCCTCGATGCAGGGCTTGTACCCCGGCGGGGGGGGCATGGCGGGC
Dog             GGCGCTTCCTTCGCCGCCTCGATGCAGGGCTTGTACCCCGGCGGGGGGGGCATGGCGGGC
Cow             GGCGCTTCCTTCGCCGCCTCGATGCAGGGCTTGTACCCCGGCGGGGGGGGCATGGCGGGC
Mouse           GGCGCTCCTTTCTCCGCCTCGGTGCAGGGTCTGTACTCCGGCGGGGGGGCCATGGCGGGC
Rat             GGCGCCCCCTTCTCCGCCTCGGTGCAGGGTCTGTACTCCGGCGGTGGGGGCATGGCGGGC
Opossum         AGTGCCTCTTTCGCCGCCTCCATGCCCGGCTTGTACCCCAACGGGGGGGGGATGGCGGGG
Platypus        GCCGCCGCCTTCGCCGCCTCCATGCCGGGGCTCTACGCGAGCGGGGGCGGGATGCCCGGA
Chick           ACGCCTCCCTTCGCCTCCTCCATGCCCGGGCTGTACTCCAGCGGCAGCGCCGTGCACCCC
                    *  * *** ** ****  ***  **  * *** *   ***  * *   **

Human           CAGAGCGCGGCCGGCGTCTACGCGGCCGGC
Chimp           CAGAGCGCGGCCGGCGTCTACGCGGCCGGC
Macaque         CAGAGCGCGGCCGGCGTCTACGCGGCCGGC
Dog             CAGAGTGCAGCCGGCGTCTACGCGGCCGGC
Cow             CAGAGCGCGGCCGGCGTCTACGCGGCCGGC
Mouse           CAGAGCGCGGCTGGCGTCTATGCGGCCGGC
Rat             CAGAGCGCGGCCGGCGTCTATGAGGCCGGC
Opossum         CAGAGCGCGGCTGGCGTCTACACAGCCGGC
Platypus        CAGAGCGGGGCC---GTGTACCCGGGCGGC
Chick           CAACCCCCCGGC---ATGTACTCCTCGGGG
                **       *      * **       **
```

13. B7-2

```
Human           GGGCTCGAGCCGAGTTCCTTCAACATGCACTGCGCGCCCTTTGAGCAGAACCTCTCCGGG
Chimp           GGGCTCGAGCCGAGTTCCTTCAACATGCACTGCGCGCCCTTTGAGCAGAACCTCTCCGGG
Macaque         GGGCTCGAGCCGAGTTCCTTCAACATGCACTGCGCGCCCTTTGAGCAGAACCTCTCCGGG
Dog             GGGCTCGAGCCGAGTTCCTTCAACATGCACTGCGCGCCCTTTGAGCAGAACCTCTCCGGG
Cow             GGGCTCGAGCCGAGTTCCTTCAACATGCACTGCGCGCCCTTTGAGCAGAACCTCTCCGGG
Mouse           GGGCTCGAACCGAGTTCCTTCAACATGCACTGCGCGCCCTTTGAGCAGAACCTCTCCGGG
Rat             GGGCTCGAACCGAGTTCCTTCAACATGCACTGCGCGCCCTTTGAGCAGAACCTCTCCGGG
Opossum         GGGCTGGAGGCGGGTTCCTTCAACATGCACTGCGCGCCCTTTGAGCAAAATCTCTCCGTG
Platypus        GGGCTGGAGGCCAGCTCCTTCAACATGCACTGCGCGCCCTTTGAGCCCAACCTCTCCGTG
Chick           GGCCTGGAGCCCGGCTCCTTCAACATGCACTGTTCCCCCTTTGAGCAAAACCTCTCCCAT
                ** ** **  *  * *****************  * **********  ** ******

Human           GTGTGTCCCGGC---GACTCCGCCAAGGCGGCGGGCGCCAAG---GAGCAGAGGGACTCG
Chimp           GTGTGTCCCGGC---GACTCCGCCAAGGCGGCGGGCGCCAAG---GAGCAGAGGGACTCG
Macaque         GTGTGTCCCGGC---GACTCCGCCAAGGCGGCGGGCGCCAAG---GAGCAGAGGGACTCG
Dog             GTGTGTCCCGGC---GACTCCGCCAAGGCGGCGGGCGCCAAG---GAGCAGAGGGACTCG
Cow             GTGTGTCCCGGC---GACTCTGCCAAGGCGGCGGGCGCCAAG---GAGCAGAGGGACTCG
Mouse           GTGTGTCCGGGC---GACCCCGCCAAGGCGGCTGGCGCCAAG---GAGCAGAGGGACTCG
Rat             GTGTGTCCGGGC---GACCCCGCCAAGGCGGCTGGCGCCAAG---GAGCAGAGGGACTCG
Opossum         ATGTGTCCCGGC---GACTCTGCCAAGGCAGGCAGCGGCAAG---GACCAGAGGGAGTCC
Platypus        ATGGGTCCCGGCGGCGACTCCGCCAAGCCCGACTGCAGCAAGGCGGGCCCCAGGGAGTCG
Chick           GATGTGCCCGGG------------AGATGCCTCCAAGCAAAA---CTGCAACACAAC---
                      ** **             *              **       *      *

Human           GACTTGGCGGCCGAGAGTAACTTCCGGATCTACCCCTGGATGCGAAGCTCA
Chimp           GACTTGGCGGCCGAGAGTAACTTCCGGATCTACCCCTGGATGCGAAGCTCA
Macaque         GACTTGGCGGCCGAGAGTAACTTCCGGATCTACCCCTGGATGCGAAGCTCA
Dog             GACTTGGCGGCCGAGAGTAACTTCCGGATCTACCCCTGGATGCGAAGCTCA
Cow             GACTTGGCGGCCGAGAGTAACTTCCGGATCTACCCCTGGATGCGAAGCTCA
Mouse           GACTTGGCGGCCGAGAGTAACTTCCGGATCTACCCCTGGATGCGAAGCTCA
Rat             GACTTGGCGGCCGAGAGTAACTTCCGGATCTACCCCTGGATGCGAAGCTCA
Opossum         GACTTGGCCGCGGAAAGTAATTTCCGGATCTACCCCTGGATGCGGAGCACA
Platypus        GACCTGGCCGGGGACAGTAACTTTCGCATCTATCCCTGGATGAGGAGCACA
Chick           ------GGACCAGAGGGA---TTCAGATTT---------------GCAAAG
                      *     **  *    **  *  *
```

14. B8-1

```
Human           ATGAGCTCTTATTTCGTCAACTCACTGTTCTCCAAATACAAAACCGGGGAGTCCCTGCGC
Chimp           ------------------------------------------------------------
Macaque         ATGAGCTCTTATTTCGTCAACTCACTGTTCTCCAAATACAAAACCGGGGAGTCCCTGCGC
Dog             ATGAGCTCTTATTTCGTCAACTCACTGTTCTCCAAATACAAAACCGGGGAGTCCCTGCGC
Cow             ATGAGCTCTTATTTCGTCAACTCACTGTTCTCCAAATACAAAACCGGGGAGTCCCTGCGC
Mouse           ATGAGCTCTTATTTCGTCAACTCACTGTTCTCCAAATACAAAACCGGGGAGTCCCTGCGC
Rat             ATGAGCTCTTATTTCGTCAACTCACTGTTCTCCAAATACAAAACCGGGGAGTCCCTGCGC
Platypus        ATGAGCTCCTATTTCGTCAACTCACTGTTCTCCAAGTACAAAACCGGGGATTCTCTACGC
Opossum         ------------------------------------------------------------
Chick           ATGAGCTCCTATTTTGTCAACTCACTCTTCTCCAAATACAAAACCGGGGACTCGTTGCGT


Human           CCCAATTATTATGACTGCGGCTTCGCCCAGGACCTGGGCGGCCGACCCACCGTGGTGTAC
Chimp           ------------------------------------------------------------
Macaque         CCCAATTATTATGACTGCGGCTTCGCCCAGGACTTGGGCGGCCGACCCACCGTGGTGTAC
Dog             CCCAATTATTATGACTGCGGCTTCGCCCAGGACCTGGGCGGCCGACCCACCGTGGTATAC
Cow             CCCAATTATTATGACTGCGGCTTCGCCCAGGACCTGGGCGGCCGACCCACCGTGGTGTAC
Mouse           CCCAATTATTATGACTGCGGCTTCGCCCAGGACCTGGGCGGCCGACCCACCGTGGTGTAC
Rat             CCCAATTATTATGACTGCGGCTTCGCCCAGGACCTGGGCGGCCGACCCACCGTGGTGTAC
Platypus        CCCAACTACTACGACTGTGGGTTCGCCCAGGACCTAGGGGGAAGACCCACCGTGGTCTAT
Opossum         ------------------------------------------------------------
Chick           CCCAATTACTATGACTGCGGGTTCGCTCAGGATCTTGGGGGCAGACCCACGGTGGTGTAC


Human           GGTCCCAGCAGCGGCGGCAGCTTCCAGCAC
Chimp           ------------------------------
Macaque         GGTCCCAGCAGCGGCGGCAGCTTCCAGCAC
Dog             GGTCCCAGCAGCGGCGGCAGCTTCCAGCAC
Cow             GGTCCCAGCAGCGGCGGTAGCTTCCAGCAC
Mouse           GGTCCCAGCAGCGGCGGCAGCTTCCAGCAC
Rat             GGTCCCAGCAGCGGCGGCAGCTTCCAGCAC
Platypus        GGCCCTAGTACCGGAGGTACCTTCCAGCAT
Opossum         ------------------------------
Chick           GGACCCAGCACGGGGGGCACCTTCCAGCAT
```

15. B8-2

```
Human           TCCGAGCAGAGCCCGTCGCCCACACAGCTCTTCCCCTGGATGCGCCCGCAAGCAGCCGCC
Chimp           ------------------------------------------------------------
Macaque         TCCGAGCAGAGTCCGTCGCCCACACAGCTCTTCCCCTGGATGCGCCCGCAAGCAGCCGCC
Dog             TCACAGCAGAGCCCGTCGCCCACACAGCTCTTCCCCTGGATGCGCCCGCAAGCAGCCGCC
Cow             TCCGAGCAGAGCCCGTCGCCCACACAGCTCTTCCCCTGGATGCGCCCGCAAGCAGCCGCC
Mouse           TCTGAGCAGAGCCCGTCGCCCACACAGCTCTTTCCCTGGATGCGCCCTCAAGCAGCCGCC
Rat             TCTGAGCAGAGCCCGTCGCCCACACAGCTCTTCCCCTGGATGCGCCCTCAAGCAGCCGCC
Platypus        TCGGAGCAGAGCCCTTCCCCGACCCAGCTCTTTCCGTGGATGAGACCGCAA---------
Opossum         ------------------------------------------------------------
Chick           TCGGAGCAGAGCCCTTCTCCGACCCAGCTTTTCCCCTGGATGCGACCGCAAGCCGCTGGA


Human           GGACGCAGGCGAGGCCGACAGACCTACAGCCGCTACCAGACCCTGGAGCTGGAGAAGGAG
Chimp           ------------------------------------------------------------
Macaque         GGACGCAGGCGAGGCCGACAGACCTACAGCCGCTACCAGACCCTGGAGCTGGAGAAGGAG
Dog             GGACGCAGGCGAGGCCGACAGACCTACAGCCGCTACCAGACCCTGGAGCTGGAGAAGGAG
Cow             GGACGCAGGCGAGGCCGACAGACCTACAGCCGCTACCAGACCCTGGAGCTGGAGAAGGAG
Mouse           GGACGCAGGCGAGGCCGCCAGACCTACAGTCGCTACCAGACCCTGGAGCTGGAGAAGGAG
Rat             GGACGCAGGCGAGGCCGCCAGACCTACAGCCGCTACCAGACCCTGGAGCTGGAGAAGGAG
Platypus        ------------------------------------------------------------
Opossum         ------------------------------------------------------------
Chick           ---CGGAGGAGGGGGAGGCAAACCTACAGCCGCTACCAGACGCTGGAACTGGAGAAGGAA


Human           TTCCTATTTAATCCCTATCTGACT
Chimp           ------------------------
Macaque         TTCCTATTTAATCCCTATCTGACT
Dog             TTCCTATTTAATCCCTATCTGACT
Cow             TTCCTATTTAATCCCTATCTGACT
Mouse           TTCCTATTTAATCCCTATCTGACT
Rat             TTCCTATTTAATCCCTATCTGACT
Platypus        ------------------------
Opossum         ------------------------
Chick           TTTCTATTTAATCCCTACCTGACC
```

16. B8-3

```
Human       CTGGGACTGACAGAGAGACAGGTCAAAATCTGGTTCCAGAACCGGAGGATGAAGTGGAAA
Chimp       ------------------------------------------------------------
Macaque     CTGGGACTGACAGAGAGACAGGTCAAAATCTGGTTCCAGAACCGGAGGATGAAGTGGAAA
Dog         CTGGGACTGACAGAGAGACAGGTCAAAATCTGGTTCCAGAACCGGAGGATGAAGTGGAAA
Cow         CTGGGACTGACAGAGAGACAGGTCAAAATTTGGTTCCAGAACCGGAGGATGAAGTGGAAA
Mouse       CTGGGACTGACAGAGAGACAGGTCAAAATCTGGTTCCAGAATCGGAGAATGAAGTGGAAA
Rat         CTGGGACTGACAGAAAGACAGGTCAAAATCTGGTTCCAGAATCGGAGAATGAAGTGGAAA
Platypus    ------------------------------------------------------------
Opossum     ------------------------------------------------------------
Chick       CTGGGATTGACAGAAAGGCAGGTCAAAATCTGGTTCCAGAACAGGAGGATGAAATGGAAA


Human       AAAGAGAACAACAAAGACAAGTTCCCCAGCAGCAAATGCGAGCAGGAGGAGCTGGAGAAA
Chimp       ------------------------------------------------------------
Macaque     AAAGAGAACAACAAAGACAAGTTCCCCAGTAGCAAATGCGAGCAGGAGGAGCTGGAGAAA
Dog         AAAGAGAACAACAAAGACAAGTTCCCCAGCAGCAAATGCGAGCAGGAGGAGCTGGAGAAA
Cow         AAAGAGAACAACAAAGACAAGTTCCCCAGCAGCAAATGCGAGCAGGAGGAGCTGGAGAAA
Mouse       AAGGAGAACAACAAAGACAAGTTTCCCAGCAGTAAATGCGAGCAGGAGGAGCTGGAGAAA
Rat         AAGGAGAACAACAAAGACAAGTTCCCCAGCAGTAAATGCGAGCAGGAGGAGCTGGAGAAA
Platypus    ------------------------------------------------------------
Opossum     ------------------------------------------------------------
Chick       AAGGAAAACAACAAAGACAAGTTTCCCAGCAGCAAATGCGAGCAGGAAGAACTGGAAAAA


Human       CAGAAGCTGGAGCGGGCC
Chimp       ------------------
Macaque     CAGAAGCTGGAGCGGGCC
Dog         CAGAAGCTGGAGCGGGCC
Cow         CAGAAGCTGGAGCGGGCC
Mouse       GAGAAGCTGGAGCGGGCA
Rat         GAGAAGCTGGAGCGGGCG
Platypus    ------------------
Opossum     ------------------
Chick       CAGAAAATGGAAAGAGCC
```

17. B9-1

```
Human           CGGCAGCCGGGCCACGCGGAGCACCTGGAGTTCCCCTCGTGCAGCTTCCAGCCCAAAGCG
Chimp           CGGCAGCCGGGCCACGCGGAGCACCTGGAGTTCCCCTCGTGCAGCTTCCAGCCCAAAGCG
Macaque         CGGCAGCCGGGCCACGCGGAGCACCTGGAGTTCCCCTCGTGCAGCTTCCAGCCCAAAGCG
Dog             CGGCAGCCGGGCCACGCGGAGCACCTGGAGTTCCCCTCGTGCAGCTTCCAGCCCAAAGCG
Cow             CGGCAGCCGGGCCACGCGGAGCACCTGGAGTTCCCCTCGTGCAGCTTCCAGCCCAAAGCG
Mouse           CGGCAGCCGGGCCACGCCGAGCACCTGGACTTCCCCTCGTGCAGCTTCCAGCCCAAAGCG
Rat             CGGCAGCCGGGCCACGCCGAGCACCTGGACTTCCCCTCGTGCAGCTTCCAGCCCAAAGCT
Opossum         CGCCAAGCCGGCCACGCGGAGCATCTCGAATTCCCCTCCTGTAGTTTCCAGCCCAAAGCT
Platypus        CGCCAGCCCGGCCACACCGAGCATTTAGACTTCCCATCCTGTAGTTTCCAGCCTAAAGCT
Chick           AGGCAAGCTGGGCACAATGAACATCTAGAATTCCCCTCTTGTAGTTTTCAGCCAAAACCT
                 * **  * ** ***   ** **  * ** ***** ** ** ** ** ***** *** *

Human           CCGGTGTTCGGCGCCTCCTGGGCGCCGCTGAGCCCGCACGCGTCCGGGAGCCTGCCGTCC
Chimp           CCGGTGTTCGGCGCCTCCTGGGCGCCGCTGAGCCCGCACGCGTCCGGGAGCCTGCCGTCC
Macaque         CCGGTGTTCGGCGCCTCCTGGGCGCCGCTGAGCCCGCACGCGTCCGGGAGCCTCCCGTCG
Dog             CCGGTGTTCGGCGCCTCCTGGGCGCCGCTGAGCCCGCACGCGTCCGGGAGCCTGCCGTCC
Cow             CCGGTGTTCGGCGCATCCTGGGCGCCGCTGAGCCCGCACGCGTCCGGAAGCCTGCCGTCC
Mouse           CCGGTGTTCGGCGCCTCCTGGGCGCCGCTGAGCCCGCACGCCTCGGGGAGCCTGCCGTCT
Rat             CCGGTGTTCGGCGCCTCCTGGGCGCCGCTGAGCCCGCACGCTTCGGGGAGCCTGCCGTCG
Opossum         CCTGTCTTCAGCGCCTCGTGGACACCTCTGAGCCCTCACTCGTCGGGGAGTCTCCCTTCT
Platypus        CCGGTGTTCAGCGCCTCGTGGACGCCCCTGAACCCCCACCCTCCGGGGACCCTCCCTTCG
Chick           CCAGTTTTCAGCGCCTCCTGGACTCCTTTGAATCCACATTCTTCTGGTACCCTTCCTGCC
                ** ** *** **** ** *** * **  ***  ** **  *  * ** *  ** **  *

Human           GTCTACCAC
Chimp           GTCTACCAC
Macaque         GTCTACCAC
Dog             GTCTACCAC
Cow             GTCTACCAC
Mouse           GTCTACCAC
Rat             GTCTACCAC
Opossum         GTCTACCAC
Platypus        GTGTATCAT
Chick           GTCTACCAT
                ** ** **
```

18. B9-2

```
Human          CTGTCTAATCAAAGACCCGGCTACGGGGACAATAAAATTTGCGAAGGAAGCGAGGACAAA
Chimp          CTGTCTAATCAAAGACCGGGCTACGGGGACAATAAAATTTGCGAAGGAAGCGAGGACAAA
Macaque        CTGTCTAATCAAAGACCCGGCTACGGGGACAATAAAATTTGCGAAGGAAGCGAGGACAAA
Dog            CTGTCTAATCAAAGACCCGGCTACGGGGACAATAAAATTTGCGAAGGAAGCGAGGACAAA
Cow            CTGTCTAATCAAAGACCCGGCTACGGGGACAATAAAATTTGCGAAGGAAGCGAGGACAAA
Mouse          CTGTCTAATCAAAGAGCTGGCTACGGGGACAATAAAATTTGCGAAGGAAGCGAGGACAAA
Rat            CTGTCTAATCAAAGAGCCGGCTACGGGGACAATAAAATTTGCGAAGGAAGCGAGGACAAA
Opossum        CTATCTAATCAAAGACCCGGCTACGGAGACAATAAACTTTGTGAAGGAAGCGAAGACAAA
Platypus       CTGTCGAATCAAAGGCCCGGCTACGAGGACAATAAAATTTGCGAAGGAAGCGAGGACAAA
Chick          GTTTCCAATCAAAGGCCCAGCTTCGAGGACAATAAAGTTTGTGAAGGAAGCGAAGACAAA
                * ** ********  *  *** **  ********* **** *********** ******

Human          GAGAGGCCGGATCAAACCAACCCCTCCGCCAACTGGCTGCACGCTCGCTCTTCCCGGAAA
Chimp          GAGAGGCCGGATCAAACCAACCCCTCCGCCAACTGGCTGCACGCTCGCTCTTCCCGGAAA
Macaque        GAGAGGCCGGATCAAACCAACCCCTCCGCCAACTGGCTGCACGCTCGCTCCTCCCGGAAA
Dog            GAGAGGCCGGATCAAACCAACCCCTCTGCCAACTGGCTGCACGCTCGCTCTTCCCGGAAA
Cow            GAGAGGCCGGATCAAACCAACCCCTCCGCCAACTGGCTGCACGCTCGCTCTTCCCGGAAA
Mouse          GAGAGGCCGGATCAAACCAACCCCTCTGCCAACTGGCTGCACGCCCGCTCTTCCCGGAAA
Rat            GAGAGGCCGGATCAAACCAACCCCTCTGCCAACTGGCTGCACGCCCGCTCTTCCCGGAAA
Opossum        GAGAGGCCTGATCAAACCAACCCCTCTGCCAACTGGTTGCATGCCCGCTCCTCCAGGAAA
Platypus       GAAAGAGCTGATCAAACCAACCCATCGGCCAGCTGGCTGCACGCTCGTTCGTCCAGGAAA
Chick          GACAGAACAGATCAAACCAACCCAGCTGCCAGCTGGCTGCACGCCCGCTCCTCCAGGAAA
               ** **  * **************  * **** **** **** ** ** ** *** *****

Human          AAGCGCTGTCCCTACACCAAATACCAGACGCTGGAGCTAGAGAAGGAGTTTCTGTTCAAT
Chimp          AAGCGCTGTCCCTACACCAAATACCAGACGCTGGAGCTAGAGAAGGAGTTTCTGTTCAAT
Macaque        AAGCGCTGTCCCTACACCAAATACCAGACGCTGGAGCTAGAGAAGGAGTTTCTGTTCAAT
Dog            AAGCGCTGTCCCTACACCAAATACCAGACGCTGGAGCTAGAGAAGGAGTTTCTGTTCAAT
Cow            AAGCGCTGTCCCTACACCAAATACCAGACGCTGGAGCTAGAGAAGGAGTTTCTGTTCAAT
Mouse          AAGCGCTGTCCCTACACCAAATACCAGACGCTGGAGCTAGAGAAGGAGTTTCTGTTCAAT
Rat            AAGCGCTGTCCCTACACCAAATACCAGACGCTGGAGCTAGAGAAGGAGTTTCTATTCAAT
Opossum        AAGCGATGCCCCTACACCAAATACCAGACGCTGGAATTAGAGAAGGAGTTTCTGTTCAAT
Platypus       AAGCGGTGTCCCTACACCAAGTACCAGACGCTGGAGTTAGAGAAGGAATTTCTGTTCAAC
Chick          AAGCGATGCCCTTACACGAAATACCAGACCCTGGAACTAGAGAAGGAGTTTTTATTCAAT
               ***** ** ** ***** ** ******** *****  ********** *** * *****

Human          ATGTACCTCACCAGGGACCGTAGGCACGAAGTGGCCAGACTCCTCAATCTGAGTGAGAGA
Chimp          ATGTACCTCACCAGGGACCGTAGGCACGAAGTGGCCAGACTCCTCAATCTGAGTGAGAGA
Macaque        ATGTACCTCACCAGGGACCGTAGGCACGAAGTGGCCAGACTCCTCAATCTGAGTGAGAGA
Dog            ATGTACCTCACCAGGGACCGTAGGCACGAAGTGGCCAGACTCCTCAATCTGAGTGAGAGA
Cow            ATGTACCTCACCAGGGACCGTAGGCACGAAGTGGCCAGACTCCTCAATCTGAGTGAGAGA
Mouse          ATGTACCTCACCAGGGACCGCAGGCATGAAGTGGCCAGACTCCTCAATCTGAGTGAGAGA
Rat            ATGTACCTCACCAGGGACCGCAGGCACGAAGTGGCCAGACTCCTCAATCTGAGTGAGAGA
Opossum        ATGTACCTCACCAGGGACCGTAGGCACGAGGTGGCCAGACTCCTCAACTTGAGTGAGAGA
Platypus       ATGTACCTCACCAGAGACCGTAGGCACGAGGTAGCCAGACTGCTCAACCTGAGCGAGAGA
Chick          ATGTACCTGACGAGGGACCGTAGACACGAAGTGGCCAGACTCCTCAACCTGAGTGAGAGA
               ******** ** ** ***** ** ** ** ** ******** *****  **** ******

Human          CAAGTCAAAATCTGGTTTCAGAACCGGCGGATGAAAATGAAGAAAATGAATAAG
Chimp          CAAGTCAAAATCTGGTTTCAGAACCGGCGGATGAAAATGAAGAAAATGAATAAG
Macaque        CAAGTCAAAATCTGGTTTCAGAACCGGCGGATGAAAATGAAGAAAATGAATAAG
Dog            CAAGTCAAAATCTGGTTTCAGAACCGGCGGATGAAAATGAAGAAAATGAATAAG
Cow            CAAGTCAAAATCTGGTTTCAGAACCGGCGGATGAAAATGAAGAAAATGAATAAG
Mouse          CAAGTCAAAATCTGGTTTCAGAACCGGCGGATGAAAATGAAGAAGATGAATAAG
Rat            CAAGTCAAAATCTGGTTTCAGAACCGGCGGATGAAAATGAAGAAGATGAATAAG
Opossum        CAAGTCAAAATCTGGTTTCAGAACCGGCGGATGAAAATGAAGAAAATGAATAAG
Platypus       CAAGTCAAAATCTGGTTTCAAAACCGAAGGATGAAAATGAAGAAAATGAACAAG
Chick          CAAGTCAAAATCTGGTTTCAGAACCGAAGGATGAAAATGAAGAAAATGAACAAG
               ******************** *****  **************** ***** ***
```

19. C4-1

```
Human         ATGATCATGAGCTCGTATTTGATGGACTCTAACTACATCGATCCGAAATTTCCTCCATGC
Chimp         ATGATCATGAGCTCGTATTTGATGGACTCTAACTACATCGATCCGAAATTTCCTCCATGC
Macaque       ATGATCATGAGCTCGTATTTGATGGACTCTAACTACATCGATCCGAAATTTCCTCCATGC
Dog           ATGATCATGAGCTCGTATTTGATGGACTCTAACTACATCGATCCGAAATTTCCTCCATGC
Cow           ATGATCATGAGCTCGTATTTGATGGACTCTAACTACATCGATCCGAAATTTCCTCCATGC
Mouse         ATGATCATGAGCTCGTATTTGATGGACTCTAACTACATCGATCCGAAATTTCCTCCATGC
Rat           ATGATCATGAGCTCGTATTTGATGGACTCTAACTACATCGATCCGAAATTTCCTCCATGC
Platypus      ------------------------------------------------------------
Opossum       ------------------------------------------------------------
Chick         ------------------------------------------------------------


Human         GAAGAATATTCGCAAAATAGCTACATCCCTGAACACAGTCCGGAATATTACGGCCGGACC
Chimp         GAAGAATATTCGCAAAATAGCTACATCCCTGAACACAGTCCGGAATATTACGGCCGGACC
Macaque       GAAGAATATTCGCAAAATAGCTACATCCCTGAACACAGTCCGGAATATTACGGCCGGACC
Dog           GAAGAATATTCGCAAAATAGCTACATCCCTGAACACAGTCCGGAATATTACGGCCGGACC
Cow           GAAGAATATTCGCAAAATAGCTACATCCCTGAACACAGTCCGGAATATTACGGCCGGACC
Mouse         GAAGAATATTCGCAAAATAGCTACATCCCTGAACACAGTCCGGAATATTACGGCCGGACC
Rat           GAAGAATATTCGCAAAATAGCTACATCCCTGAACACAGTCCGGAATATTACGGCCGGACC
Platypus      ------------------------------------------------------------
Opossum       ------------------------------------------------------------
Chick         ------------------------------------------------------------


Human         AGGGAATCGGGATTCCAGCATCACCACCAGGAGCTGTACCCACCACCGCCTCCGCGCCCT
Chimp         AGGGAATCGGGATTTCAGCATCACCACCAGGAGCTGTACCCACCACCGCCTCCGCGCCCT
Macaque       AGGGAATCGGGATTCCAGCATCACCACCAGGAGCTGTACCCACCACCGCCTCCGCGCCCT
Dog           AGGGAATCGGGATTCCAGCATCACCACCAGGAGCTGTACCCACCACCGCCTCCGCGCCCT
Cow           AGGGAATCGGGATTCCAGCATCACCACCAGGAGCTGTACCCACCACCGCCTCCGCGCCCT
Mouse         AGGGAATCGGGATTCCAGCATCACCACCAGGAGCTGTACCCACCACCGCCTCCGCGCCCT
Rat           AGGGAATCGGGATTCCAGCATCACCACCAGGAGCTGTACCCACCACCGCCTCCGCGCCCT
Platypus      ------------------------------------------------------------
Opossum       ------------------------------------------------------------
Chick         ------------------------------------------------------------


Human         AGCTACCCTGAGCGCCAGTATAGCTGCACCAGTCTCCAGGGG
Chimp         AGCTACCCTGAGCGCCAGTATAGCTGCACCAGTCTCCAGGGG
Macaque       AGCTACCCTGAGCGCCAGTATAGCTGCACCAGTCTCCAGGGG
Dog           AGCTACCCCGAGCGCCAGTATAGCTGCACCAGTCTCCAGGGG
Cow           AGCTACCCTGAGCGCCAGTATAGCTGCACCAGTCTCCAGGGG
Mouse         AGCTACCCTGAGCGTCAGTATAGCTGCACCAGTCTCCAGGGG
Rat           AGCTACCCTGAGCGTCAGTATAGCTGCACCAGTCTCCAGGGG
Platypus      ------------------------------------------
Opossum       ------------------------------------------
Chick         ------------------------------------------
```

20. C4-2

```
Human          GGCAATTCGCGAGGCCACGGGCCGGCCCAGGCGGGCCACCACCACCCCGAGAAATCACAG
Chimp          GGCAATTCGCGAGGCCACGGGCCGGCCCAGGCGGGCCACCACCACCCCGAGAAATCACAG
Macaque        GGCAATTCGCGAGGCCACGGGCCGGCCCAGGCGGGCCACCACCACCCCGAGAAATCACAG
Dog            GGCAGTTCGCGAGGCCACGGGCCGGCCCAGGCGGGCCACCACCACCCCGAGAAATCCCAG
Cow            GGCAATTCGCGAGGCCACGGGCCGGCCCAGGCGGGCCACCACCACCCCGAGAAATCACAG
Mouse          GGCAATTCGCGAGGCCACGGGCCGGCCCAGGCGGGCCACCACCACCCTGAGAAATCACAG
Rat            GGCAATTCGCGAGCCCACGGGCCGGCCCAGGCGGGCCACCACCACCCTGAGAAATCGCAG
Platypus       ------------------------------------------------------------
Opossum        ------------------------------------------------------------
Chick          ------------------------------------------------------------


Human          TCGCTCTGCGAGCCGGCGCCTCTCTCAGGCGCCTCCGCCTCCCCGTCCCCAGCCCCGCCA
Chimp          TCGCTCTGCGAGCCGGCGCCTCTCTCAGGCGCCTCCGCCTCCCCGTCCCCAGCCCCGCCA
Macaque        TCGCTCTGCGAGCCGGCGCCTCTCTCAGGCGCCTCCGCCTCCCCGTCCCCAGCCCCGCCA
Dog            CCGCTCTGCGAGCCGGCGCCTCTCTCGGGCGCCTCCGCCTCCCCGTCCCCAGCCCCGCCA
Cow            CCGCTCTGCGAGCCGGCGCCTCTCTCAGGCGCCTCCGCCTCCCCGTCCCCAGCCCCGCCA
Mouse          TCGCTCTGCGAGCCGGCGCCTCTCTCAGGCACCTCTGCCTCCCCGTCCCCAGCCCCGCCA
Rat            CCGCTCTGCGAGCCGGCGCCTCTCTCAGGCGCCTCTGCCTCCCCGTCCCCAGCCCCGCCA
Platypus       ------------------------------------------------------------
Opossum        ------------------------------------------------------------
Chick          ------------------------------------------------------------


Human          GCCTGCAGCCAGCCAGCC
Chimp          GCCTGCAGCCAGCCAGCC
Macaque        GCCTGCAGCCAGCCAGCC
Dog            GCCTGCAGCCAGCCAGCC
Cow            GCCTGCAGCCAGCCAGCC
Mouse          GCCTGCAGCCAGCCGGCC
Rat            GCCTGCAGCCAGCCAGCC
Platypus       ------------------
Opossum        ------------------
Chick          ------------------
```

21. C4-3

```
Human          GTCCTGGAATTAGAGAAAGAGTTTCATTACAACCGCTACCTGACCCGAAGGAGAAGGATC
Chimp          GTCCTGGAATTAGAGAAAGAGTTTCATTACAACCGCTACCTGACCCGAAGGAGAAGGATC
Macaque        GTCCTGGAATTAGAGAAAGAGTTTCATTACAACCGCTACCTGACCCGAAGGAGAAGGATC
Dog            GTCCTGGAATTAGAGAAAGAGTTTCATTACAATCGCTACCTGACCCGAAGGAGAAGGATC
Cow            GTCCTGGAATTAGAGAAAGAGTTTCATTACAATCGCTACCTGACCCGAAGGAGAAGGATC
Mouse          GTCCTGGAATTAGAGAAAGAGTTTCATTACAACCGCTACCTGACCCGAAGGAGAAGGATC
Rat            GTCCTGGAATTAGAGAAAGAGTTTCATTACAACCGCTACCTGACCCGAAGGAGAAGGATC
Platypus       ------------------------------------------------------------
Opossum        ------------------------------------------------------------
Chick          ------------------------------------------------------------


Human          GAGATCGCCCACTCGCTGTGCCTCTCTGAGAGGCAGATCAAAATCTGGTTCCAAAACCGT
Chimp          GAGATCGCCCACTCGCTGTGCCTCTCTGAGAGGCAGATCAAAATCTGGTTCCAAAACCGT
Macaque        GAGATCGCCCACTCGCTGTGCCTCTCTGAGAGGCAGATCAAAATCTGGTTCCAAAACCGT
Dog            GAGATCGCCCACTCGCTGTGCCTCTCTGAGAGGCAGATCAAAATCTGGTTTCAAAACCGG
Cow            GAGATCGCCCACTCGCTGTGCCTCTCTGAGAGGCAGATCAAAATCTGGTTCCAAAACCGT
Mouse          GAGATCGCCCACTCGCTGTGCCTCTCTGAGAGGCAGATCAAAATCTGGTTCCAAAACCGT
Rat            GAGATCGCCCACTCGCTGTGCCTCTCTGAGAGGCAGATCAAAATCTGGTTCCAAAACCGT
Platypus       ------------------------------------------------------------
Opossum        ------------------------------------------------------------
Chick          ------------------------------------------------------------
```

22. C5

```
Human           ATGAGCTCCTACGTAGCCAATTCATTCTATAAGCAGAGCCCCAATATCCCTGCCTATAAC
Chimp           ATGAGCTCCTACGTAGCCAATTCATTCTATAAGCAGAGCCCCAATATCCCTGCCTATAAC
Macaque         ATGAGCTCCTACGTAGCCAATTCATTCTATAAGCAGAGCCCCAATATCCCTGCCTATAAC
Dog             ATGAGCTCCTACGTAGCCAATTCATTCTATAAGCAGAGCCCCAATATCCCTGCCTATAAC
Cow             ATGAGCTCCTACGTAGCCAATTCATTCTATAAGCAGAGCCCCAATATCCCTGCCTATAAC
Mouse           ATGAGCTCCTACGTAGCCAATTCATTCTATAAGCAGAGCCCCAATATCCCTGCCTATAAC
Rat             ATGAGCTCCTACGTAGCCAATTCATTCTATAAGCAGAGCCCCAATATCCCTGCCTATAAC
Platypus        ------------------------------------------------------------
Opossum         ------------------------------------------------------------
Chick           ------------------------------------------------------------


Human           ATGCAAACTTGTGGGAACTATGGATCGGCCTCAGAGGTGCAGGCATCCAGGTACTGCTAC
Chimp           ATGCAAACTTGTGGGAACTATGGATCGGCCTCAGAGGTGCAGGCATCCAGGTACTGCTAC
Macaque         ATGCAAACTTGTGGGAACTATGGATCGGCCTCAGAGGTGCAGGCATCCAGGTACTGCTAC
Dog             ATGCAAACTTGTGGGAACTATGGATCGGCCGCAGAGGTGCAGGCATCCAGGTACTGCTAC
Cow             ATGCAAACTTGTGGGAACTATGGATCGGCCTCAGAGGTGCAGGCATCCAGGTACTGCTAC
Mouse           ATGCAAACTTGTGGGAACTATGGATCGGCCTCAGAGGTGCAGGCATCCAGGTACTGCTAC
Rat             ATGCAAACTTGTGGGAACTATGGATCGGCCTCAGAGGTGCAGGCATCCAGGTACTGCTAC
Platypus        ------------------------------------------------------------
Opossum         ------------------------------------------------------------
Chick           ------------------------------------------------------------


Human           GGCGGATTGGACTTAAGCATCACTTTCCCACCGCCTGCGCCTTCCAACTCTCTCCACGGG
Chimp           GGCGGATTGGACTTAAGCATCACTTTCCCACCGCCTGCGCCTTCCAACTCTCTCCACGGG
Macaque         GGCGGATTGGACTTAAGCATCACTTTCCCACCGCCTGCGCCTTCCAACTCTCTCCACGGG
Dog             GGTGGATTGGACTTAAGCATCACTTTCCCACCGCCTGCGCCTTCCAACTCTCTCCACGGG
Cow             GGCGGATTGGACTTAAGCATCACTTTCCCACCGCCTGCGCCTTCCAACTCTCTCCACGGG
Mouse           GGCGGATTGGACTTAAGCATCACTTTCCCACCGCCTGCGCCTTCCAACTCTCTCCACGGG
Rat             GGCGGATTGGACTTAAGCATCACTTTCCCACCGCCTGCGCCTTCCAACTCTCTCCACGGG
Platypus        ------------------------------------------------------------
Opossum         ------------------------------------------------------------
Chick           ------------------------------------------------------------


Human           GTAGACATGGCTGCCAACCCCCGGGCTCACCCCGACCGCCCCGCCTGCAGCGCCGCGGCC
Chimp           GTAGACATGGCTGCCAACCCCCGGGCTCACCCCGACCGCCCCGCCTGCAGCGCCGCGGCC
Macaque         GTAGACATGGCTGCCAACCCCCGGGCTCACCCCGACCGCCCCGCCTGCAGCGCCGCGGCC
Dog             GTAGACATGGCAGCCAGCCCCCGGGCTCACCCCGACCGCCCCGCCTGCAGCGCCGCGGCC
Cow             GTAGACATGGCTGCCAACCCCCGGGCTCACCCCGACCGCCCCGCCTGCAGCGCCGCAGCC
Mouse           GTAGACATGGCTGCCAACCCCCGGGCTCACCCCGACCGCCCCGCCTGCAGCGCCGCGGCC
Rat             GTAGACATGGCTGCCAACCCCCGGGCTCACCCCGACCGCCCCGCCTGCAGCGCTGCGGCC
Platypus        ------------------------------------------------------------
Opossum         ------------------------------------------------------------
Chick           ------------------------------------------------------------


Human           GCTCCGGGACACGCTCCGGGCAGAGACGAAGCGGCTCCTCTGAACCCCGGGATGTACAGT
Chimp           GCTCCGGGACACGCTCCGGGCAGAGACGAAGCGGCTCCTCTGAACCCCGGGATGTACAGT
Macaque         GCTCCGGGACACGCTCTGGGCAGAGACGAAGCGGCTCCTCTGAACCCCGGGATGTACAGT
Dog             GCTCCGGGACACGCTCTGGGCAGAGACGAAGCGGCTCCTCTGAACCCCGGGATGTACAGT
Cow             GCTCCGGGACACGCTCTGGGCAGAGATGAAGCGGCTCCTCTGAACCCCGGGATGTACAGT
Mouse           GCTCCGGGACACGCTCTGGGCAGAGACGAAGCGGCTCCTCTGAACCCTGGGATGTACAGT
Rat             GCTCCGGGACACGCTCTGGGCAGAGACGAAGCGGCTCCTCTGAACCCTGGGATGTACAGT
Platypus        ------------------------------------------------------------
Opossum         ------------------------------------------------------------
Chick           ------------------------------------------------------------


Human           CAGAAGGCGGCTCGCCCGGCGCTGGAGGAGCGAGCTAAGAGCAGTGGGGAGATCAAAGAG
```

```
Chimp           CAGAAGGCGGCTCGCCCGGCGCTGGAGGAGCGAGCTAAGAGCAGTGGGGAGATCAAAGAG
Macaque         CAGAAGGCGGCTCGCCCGGCGCTGGAGGAGCGAGCTAAGAGCAGTGGGGAGATCAAAGAG
Dog             CAGAAGGCGGCTCGCCCAGCGCCGGAGGAGCGAGCTAGGAGCACTGGGGAGATCAAAGAG
Cow             CAGAAGGCGGCTCGCCCAGCGCTGGAGGAGCGAGCTAAGAGCAGTGGGGAGATCAAAGAG
Mouse           CAGAAGGCGGCTCGCCCGGCGCTGGAGGAGCGAGCTAAGAGCAGTGGGGAGATCAAAGAG
Rat             CAGAAGGCGGCCCGCCCGGCGCTGGAGGAGCGAGCTAAGAGCAGTGGGGAGATCAAAGAG
Platypus        ------------------------------------------------------------
Opossum         ------------------------------------------------------------
Chick           ------------------------------------------------------------


Human           GAGCAGGCGCAGACAGGGCAGCCCGCCGGACTGAGCCAGCCACCGGCCCCGCCACAGATT
Chimp           GAGCAGGCGCAGACAGGGCAGCCCGCCGGACTGAGCCAGCCACCGGCCCCGCCACAGATT
Macaque         GAGCAGGCGCAGACAGGGCAGCCCGCCGGACTGAGCCAGCCACCGGCCCCGCCACAGATT
Dog             GAGCAGGCGCAGACAGGGCAGCCCGCCGCCCGGAGCCAGCCACCGGCCCCGCCACAGATT
Cow             GAGCAGGCGCAGACAGGGCAGCCTGCCGGACTGAGCCAGCCACCGGCCGCGCCACAGATT
Mouse           GAGCAGGCGCAGACAGGGCAGCCTGCCGGACTGAGCCAGCCACCGGCCCCGCCACAGATT
Rat             GAGCAGGCGCAGACAGGGCAGCCTGCCGGACTGAGCCAGCCACCGGCCCCGCCACAGATT
Platypus        ------------------------------------------------------------
Opossum         ------------------------------------------------------------
Chick           ------------------------------------------------------------


Human           TACCCGTGGATGACCAAACTGCACATGAGCCACGAGACGGACGGCAAGCGGTCCCGAACC
Chimp           TACCCGTGGATGACCAAACTGCACATGAGCCACGAGACGGACGGCAAGCGGTCCCGAACC
Macaque         TACCCGTGGATGACCAAACTGCACATGAGCCACGAGACGGACGGCAAGCGGTCCCGAACC
Dog             TACCCGTGGATGACCAAACTGCACATGAGCCACGAGACGGACGGCAAGCGGTCCCGAACC
Cow             TACCCGTGGATGACCAAACTGCACATGAGCCACGAGACGGACGGCAAGCGGTCCCGAACC
Mouse           TACCCGTGGATGACCAAACTGCACATGAGCCACGAGACGGATGGCAAGCGGTCCCGAACC
Rat             TACCCGTGGATGACCAAACTGCACATGAGCCACGAGACGGATGGCAAGCGGTCCCGAACC
Platypus        ------------------------------------------------------------
Opossum         ------------------------------------------------------------
Chick           ------------------------------------------------------------


Human           AGTTACACGCGCTACCAGACTCTGGAA
Chimp           AGTTACACGCGCTACCAGACTCTGGAA
Macaque         AGTTACACGCGCTACCAGACTCTGGAA
Dog             AGTTACACGCGCTACCAGACTCTGGAA
Cow             AGTTACACGCGCTACCAGACTCTGGAA
Mouse           AGTTACACGCGCTACCAGACCCTGGAA
Rat             AGTTACACGCGCTACCAGACCCTGGAA
Platypus        ---------------------------
Opossum         ---------------------------
Chick           ---------------------------
```

23. C6

```
Human           ATGAATTCCTACTTCACTAACCCTTCCTTATCCTGCCACCTCGCCGGGGGCCAGGACGTC
Chimp           ATGAATTCCTACTTCACTAACCCTTCCTTATCCTGCCACCTCGCCGGGGGCCAGGACGTC
Macaque         ATGAATTCCTACTTCACTAACCCTTCCTTATCCTGCCACCTCGCCGGGGGCCAGGACGTC
Dog             ATGAATTCCTACTTCACTAACCCTTCCTTATCCTGCCACCTCGCCGGGGGCCAGGACGTC
Cow             ATGAATTCCTACTTCACTAACCCTTCCTTATCCTGCCACCTCGCCGGGGGCCAGGACGTC
Mouse           ATGAATTCCTACTTCACTAACCCTTCCTTATCCTGCCACCTCGCCGGGGGCCAGGACGTC
Rat             ATGAATTCCTACTTCACTAACCCTTCCTTATCCTGCCACCTCGCCGGGGGCCAGGACGTC
Opossum         ATGAATTCCTATTTCACTAACCCGTCCTTATCGTGCCATCTCGCCGGGGGTCAGGACGTT
Platypus        ATGAATTCGTATTTCACCAACCCCCCTCTGTCCTGCCATCTCGCCGCCAGTCAAGACGCG
Chick           ATGAATTCCTACTTCACTAACCCATCTTTATCCTGCCATCTAACCAGTGGCCAAGAGGTG
                ******** ** ***** *****  *  * ** ***** **  **    * ** ** *

Human           CTCCCCAACGTCGCCCTCAATTCCACCGCCTATGATCCAGTGAGGCATTTCTCGACCTAT
Chimp           CTCCCCAACGTCGCCCTCAATTCCACCGCCTATGATCCAGTGAGGCATTTCTCGACCTAT
Macaque         CTCCCCAACGTCGCCCTCAATTCCACCGCCTATGATCCAGTGAGGCATTTCTCGACCTAT
Dog             CTCCCCAACGTCGCCCTCAATTCCACCGCCTATGATCCAGTGAGGCATTTCTCGACCTAT
Cow             CTCCCCAACGTCGCCCTCAATTCCACCGCTTATGATCCAGTGAGGCATTTCTCGACCTAT
Mouse           CTCCCCAACGTCGCCCTCAATTCCACCGCCTATGATCCAGTGAGGCATTTCTCGACCTAT
Rat             CTCCCCAACGTCGCCCTCAATTCCACCGCCTATGATCCAGTGAGGCATTTCTCGACCTAT
Opossum         CTCCCCAACGTCGCCCTCAATTCCACCGCCTATGATCCAGTGAGACATTTCTCGACCTAT
Platypus        CTTCCAAACGTGGCTCTCAACAGCAGCGCCTACGACCCCGTGAGACACTTCTCCGCCTAC
Chick           CTTCCCAACGTAGCCCTCAATTCAACTGCCTATGACCCTGTCAGGCATTTTTCTACTTAT
                ** ** ***** ** *****    *  ** ** ** ** ** ** ** ** **  * **

Human           GGAGCGGCCGTTGCCCAGAACCGGATCTACTCGACTCCCTTTTATTCGCCACAGGAGAAT
Chimp           GGAGCGGCCGTTGCCCAGAACCGGATCTACTCGACTCCCTTTTATTCGCCACAGGAGAAT
Macaque         GGAGCAGCCGTTGCCCAGAACCGGATCTACTCGACTCCCTTTTATTCGCCACAGGAGAAT
Dog             GGAGCAGCCGTTGCCCAGAACCGGATCTACTCGACTCCCTTTTATTCGCCACAGGAGAAT
Cow             GGAGCGGCCGTGGCTCAGAACCGGATCTACTCGACTCCCTTTTATTCGCCACAGGAGAAT
Mouse           GGAGCAGCCGTAGCTCAGAACCGGATCTACTCGACTCCCTTTTATTCGCCACAGGAGAAT
Rat             GGAGCAGCCGTAGCTCAGAACCGGATCTACTCGACTCCCTTTTATTCGCCACAGGAGAAT
Opossum         GGAGCGGCAGTGGCACAGAACCGGATCTACTCGTCTCCCTTTTATTCGCCACAAGAGAAT
Platypus        GGGGCTGCCGTCGCCCAGAACCGGATTTATTCGCCCCCCTTTTATTCCCCCCAAGACAAC
Chick           GGAGCAGCCGTTGCTCAAAGCCGGATTTATTCTTCTCCTTTTTATTCACCGCAAGATAAT
                ** ** ** ** ** ** * ****** ** **  * ** ******** ** ** ** **

Human           GTCGTGTTCAGTTCCAGCCGGGGGCCGTATGACTATGGATCTAATTCCTTTTACCAGGAG
Chimp           GTCGTGTTCAGTTCCAGCCGGGGGCCGTATGACTATGGATCTAATTCCTTTTACCAGGAG
Macaque         GTCGTGTTCAGTTCCAGCCGGGGGCCGTATGACTATGGATCTAATTCCTTTTACCAGGAG
Dog             GTCGTGTTCAGTTCCAGCCGGGGGCCGTATGACTATGGATCTAATTCCTTTTACCAGGAG
Cow             GTCGTGTTCAGTTCCAGCCGAGGGCCGTATGACTATGGATCTAATTCCTTTTACCAGGAG
Mouse           GTCGTGTTCAGTTCCAGCCGGGGGCCGTATGACTATGGATCTAATTCCTTTTACCAGGAG
Rat             GTCGTGTTCAGTTCCAGCCGGGGGCCGTATGACTATGGATCTAATTCCTTTTACCAGGAG
Opossum         GTCGTGTTCAGTTCGAGCCGGGGGCCGTATGACTATGGATCTAATTCCTTTTACCAGGAA
Platypus        GTCGTGTTCGGCTCCGGCCGGGGGCCCTACGAGTACGGATCTAACTCCTTTTACCAGGAG
Chick           GTTGTGTTTAGCTCCAGCCGAGGACCTTATGACTATGGATCTAATGCTTTCTACCAAGAA
                ** *****  * **  **** ** ** ** ** ** ********  * ** ***** **

Human           AAAGACATGCTCTCAAACTGCAGACAAAACACCTTAGGACATAACACACAGACCTCAATC
Chimp           AAAGACATGCTCTCAAACTGCAGACAAAACACCTTAGGACATAACACACAGACCTCAATC
Macaque         AAAGACATGCTCTCAAACTGCAGACAAAACACCTTAGGACATAACACACAGACCTCAATC
Dog             AAAGACATGCTCTCAAACTGCAGACAAAACACCTTAGGACATAACACACAGACCTCAATC
Cow             AAAGACATGCTCTCAAACTGCAGACAAAACACCTTAGGACATAACACACAGACCTCAATC
Mouse           AAAGACATGCTCTCAAACTGCAGACAAAACACCTTAGGACATAACACACAGACCTCAATC
Rat             AAAGACATGCTCTCAAACTGCAGACAAAACACCTTAGGACATAACACACAGACCTCAATC
Opossum         AAAGACATGCTCTCAAATTGCAGGCAGAACACCTTAGCACACAACACACAGACCTCAATG
Platypus        AAAGACGTTCTGTCGAACTGCCGACAGAGCACCCTGGGACACAACTCACAGAACTCAATC
Chick           AAAGACATGCTTTCTAGCTGCAGGCAAAATTCTATGGGACATAATACACAGACATCAATC
                ****** * ** ** *  *** * ** *   *  * * *** **  ******  *****

Human           GCTCAGGATTTTAGTTCTGAGCAGGGCAGGACTGCGCCCCAGGACCAGAAAGCCAGTATC
```

```
Chimp           GCTCAGGATTTTAGTTCTGAGCAGGGCAGGACTGCGCCCCAGGACCAGAAAGCCAGTATC
Macaque         GCTCAGGATTTTAGTTCTGAGCAGGGCAGGACTGCGCCCCAGGACCAGAAAGCCAGTATC
Dog             GCTCAGGATTTTAGTTCTGAGCAGGGCAGGACTGCGCCCCAGGACCAGAAAGCCAGTATC
Cow             GCTCAGGATTTTAGTTCTGAGCAGGGCAGGACTGCGCCCCAGGACCAGAAAGCCAGTATC
Mouse           GCTCAGGATTTTAGTTCTGAGCAGGGCAGGACTGCGCCCCAGGACCAGAAAGCCAGTATC
Rat             GCTCAGGATTTTAGTTCTGAGCAGGGCAGGACTGCGCCCCAGGACCAGAAAGCCAGTATC
Opossum         GCCCAGGACTTTAGCTCTGAGCAAAGCAGGACTGCTCCCCAAGACCAGAAAACCAGTATC
Platypus        ACTCAGGATTTTAGCAGCGAGCAGACGAGAACGACCGGCCAGGATCAGAAAGCCAGTATC
Chick           GCACAGGATTTTACCAGTGACCAAAACAGGAACACTTCGCAAGAACAAAAAACTAGCATT
                 * ***** ****     ** **    ** *   *    ** ** ** *** * ** **

Human           CAGATTTACCCCTGGATGCAGCGAATGAATTCGCACAGTGGGGTCGGCTACGGAGCGGAC
Chimp           CAGATTTACCCCTGGATGCAGCGAATGAATTCGCACAGTGGGGTCGGCTACGGAGCGGAC
Macaque         CAGATTTACCCCTGGATGCAGCGAATGAATTCGCACAGTGGGGTCGGCTACGGAGCGGAC
Dog             CAGATTTACCCCTGGATGCAGCGAATGAATTCGCACAGTGGGGTCGGCTACGGAGCGGAC
Cow             CAGATTTACCCCTGGATGCAGCGAATGAATTCGCACAGTGGGGTCGGCTATGGAGCGGAC
Mouse           CAGATTTACCCCTGGATGCAGCGAATGAATTCGCACAGTGGGGTCGGTTACGGAGCGGAC
Rat             CAGATTTACCCCTGGATGCAGCGAATGAATTCGCACAGTGGGGTCGGTTACGGAGCGGAC
Opossum         CAGATCTATCCCTGGATGCAGCGAATGAATTCGCACAGTGGGGTCGGCTATGGGGCGGAT
Platypus        CAGATCTACCCTTGGATGCAGCGAATGAATTCGCACAGTGGAGTCGGCTACGGGGCGGAC
Chick           CAAATATACCCATGGATGCAGCGTATGAACTCCCACAGTGGCGTGGGCTACGGGGCCGAC
                ** ** ** ** *********** ***** ** ******** ** ** ** ** ** **

Human           CGGAGGCGCGGCCGCCAGATCTACTCGCGGTACCAGACCCTGGAACTGGAGAAG
Chimp           CGGAGGCGCGGCCGCCAGATCTACTCGCGGTACCAGACCCTGGAACTGGAGAAG
Macaque         CGGAGGCGCGGCCGCCAGATCTACTCGCGGTACCAGACCCTGGAACTGGAGAAG
Dog             CGGAGGCGCGGCCGCCAGATCTACTCGCGGTACCAGACCCTGGAACTGGAGAAG
Cow             CGGAGGCGCGGTCGCCAGATCTACTCGAGGTACCAGACGCTGGAACTGGAGAAG
Mouse           CGGAGGCGCGGCCGCCAGATCTACTCTCGGTACCAGACCCTGGAACTGGAGAAG
Rat             CGGAGGCGCGGCCGCCAGATCTACTCTCGGTACCAGACCCTGGAACTGGAGAAA
Opossum         AGGAGACGCGGCCGGCAGATCTACTCTCGATACCAGACCTTGGAGCTGGAAAAA
Platypus        AGGAGACGGGGTCGCCAGATTTACTCTCGCTACCAGACCCTGGAGCTGGAGAAG
Chick           CGCCGGCGGGGCCGCCAGATTTATTCCCGTTACCAAACGTTGGAGCTGGAGAAG
                 *  * ** ** ** ***** ** **  * ***** **  **** ***** **
```

24. C8

```
Human           TGCTCGCTTAGCTGCCACGGAGACGCCTCCAAATTCTATGGCTACGAGGCGCTCCCCAGA
Chimp           TGCTCGCTTAGCTGCCACGGAGACGCCTCCAAATTCTATGGCTACGAGGCGCTCCCCAGA
Macaque         TGCTCGCTTAGCTGCCACGGAGACGCCTCCAAATTCTATGGCTACGAGGCGCTCCCCAGA
Dog             TGCTCGCTGAGCTGCCACGGAGACGCCTCCAAATTCTATGGCTACGAGGCGCTCCCCAGA
Cow             TGCTCGCTGAGCTGCCACGGAGACGCCTCCAAATTCTATGGCTACGAGGCGCTCCCCAGA
Mouse           TGCTCGCTGAGCTGCCACGGAGACGCCTCCAAATTCTATGGCTACGAGGCGCTCCCCAGA
Rat             TGCTCGCTGAGCTGCCACGGAGACGCCTCCAAATTCTATGGCTACGAGGCGCTCCCCAGA
Opossum         ------------------------------------------------------------
Platypus        ------------------------------------------------------------
Chick           TGCGCCTTGGCGTGTCACGGAGACGCTTCTAAATTCTATGGATATGAAGCTCTGCCGAGG


Human           CAGTCCCTTTATGGGGCTCAGCAAGAGGCGAGCGTGGTGCAATATCCCGACTGTAAATCC
Chimp           CAGTCCCTTTATGGGGCTCAGCAAGAGGCGAGCGTGGTGCAATATCCCGACTGTAAATCC
Macaque         CAGTCCCTTTATGGGGCTCAGCAAGAGGCGAGCGTGGTGCAATATCCCGACTGTAAATCC
Dog             CAGTCCCTTTATGGGGCCCAGCAAGAGGCGAGCGTGGTGCAATATCCCGACTGTAAATCC
Cow             CAGTCCCTTTATGGGGCTCAGCAAGAGGCGAGCGTGGTGCAATATCCCGACTGTAAATCC
Mouse           CAGTCCCTTTATGGGGCTCAGCAAGAGGCGAGCGTGGTGCAATATCCCGACTGTAAATCC
Rat             CAGTCCCTTTATGGGGCTCAGCAAGAGGCGAGCGTGGTGCAATATCCCGACTGTAAATCC
Opossum         ------------------------------------------------------------
Platypus        ------------------------------------------------------------
Chick           CAATCGCTTTATGGTGCTCAGCAAGAGACGACTGTTGTACAATATCCTGACTGTAAATCG


Human           TCCGCCAACACTAACAGTAGCGAAGGACAAGGCCACTTAAATCAAAACTCGTCTCCCAGC
Chimp           TCCGCCAACACTAACAGTAGCGAAGGACAAGGCCACTTAAATCAAAACTCGTCTCCCAGC
Macaque         TCCGCCAACACTAACAGTAGCGAAGGACAAGGCCACTTAAATCAAAACTCGTCTCCCAGC
Dog             TCCGCCAACACTAACAGTAGCGAAGGACAAGGCCACTTAAATCAAAACTCGTCTCCCAGC
Cow             TCCGCCAACACTAACAGTAGCGAAGGACAAGGCCACTTAAATCAAAACTCGTCTCCCAGC
Mouse           TCCGCCAACACTAACAGTAGCGAAGGACAAGGCCACTTAAATCAGAACTCGTCTCCCAGC
Rat             TCCGCCAACACTAACAGTAGCGAAGGACAAGGCCACTTAAATCAGAACTCGTCTCCCAGC
Opossum         ------------------------------------------------------------
Platypus        ------------------------------------------------------------
Chick           TCTTCCAACAGTAACTCTAGCGAGGGACAAGGGCATTTAAATCAAAATTCGTCTCCCAGT


Human           CTCATGTTTCCATGGATGAGACCCCACGCTCCGGGG
Chimp           CTCATGTTTCCATGGATGAGACCCCACGCTCCGGGG
Macaque         CTCATGTTTCCATGGATGAGACCCCACGCTCCGGGG
Dog             CTCATGTTTCCATGGATGAGACCCCACGCTCCGGGG
Cow             CTCATGTTTCCATGGATGAGACCCCACGCTCCGGGG
Mouse           CTCATGTTTCCATGGATGAGACCCCACGCTCCTGGG
Rat             CTCATGTTTCCATGGATGAGACCCCACGCTCCTGGG
Opossum         ------------------------------------
Platypus        ------------------------------------
Chick           CTCATGTTCCCATGGATGAGACCTCACGCTCCCGGA
```

25. C9

```
Human          CAC---CCGTACGGCCCCCAGCCCCACCTCGGCGCCGACACGCGCTACATGCGGACTTGG
Chimp          CAC---CCGTACGGCCCCCAGCCCCACCTCGGCGCCGACACGCGCTACATGCGGACTTGG
Macaque        CAC---CCGTACGGCCCCCAGCCCCACCTCGGCGCCGACACGCGCTACATGCGGACTTGG
Dog            ------------------------------------------------------------
Cow            CAC---CCGTACGGCCCCCAGCCCCACCTCGGCGCCGACACGCGCTACATGCGGACTTGG
Mouse          CAC---CCTTACGGCCCCCAGCCCCACCTCGGCGCCGACACGCGCTACATGCGGACTTGG
Rat            CAC---CCTTACGGCCCCCAGCCCCACCTCGGCGCCGACACGCGCTACATGCGGACTTGG
Opossum        ------------------------------------------------------------
Platypus       CAC---CCGTACACGCACCAGCCCCACATCGGCGCCGACTCCAGGTACATGCGGACGTGG
Chick          CACCACCCGTACGGCCCCCAGGCTCCCGTGGGGGCCGAGCCCAGGTACATGCGGACTTGG


Human          CTCGAGCCGCTGTCCGGCGCCGTCTCCTTCCCCAGCTTC---CCGGCCGGGGGCCGTCAC
Chimp          CTCGAGCCGCTGTCCGGCGCCGTCTCCTTCCCCAGCTTC---CCGGCCGGGGGCCGTCAC
Macaque        CTCGAGCCGCTGTCCGGCGCCGTCTCTTTCCCCAGCTTC---CCGGCCGGGGGCCGTCAC
Dog            ------------------------------------------------------------
Cow            CTCGAGCCGCTGTCCGGCGCCGTCTCCTTCCCCAGCTTC---CCGGCCGGGGGCCGTCAC
Mouse          CTCGAGCCGCTGTCCGGCGCCGTCTCCTTCCCCAGCTTC---CCGGCCGGGGGCCGTCAC
Rat            CTCGAGCCGCTGTCCGGCGCCGTCTCCTTCCCCAGCTTC---CCAGCCGGGGGCCGTCAC
Opossum        ------------------------------------------------------------
Platypus       TTGGAACCTCTCTCGGGAGCCGTCTCGTTCCCGGGCTTC---GCGGCCGGCGGCCGCCCC
Chick          CTCGAGCCCCTCGCCGGGGCCGTCTCGTTCCCGGCCTTCGCTCCCGGTGCCGCCCGCCCC


Human          TACGCCCTCAAGCCGGACGCCTACCCCGGGCGCCGC
Chimp          TACGCCCTCAAGCCGGACGCCTACCCCGGGCGCCGC
Macaque        TACGCCCTCAAGCCGGACGCCTACCCCGGGCGCCGC
Dog            ------------------------------------
Cow            TACGCCCTCAAGCCGGACGCCTACCCCGGGCGCCGC
Mouse          TACGCCCTCAAGCCCGACGCCTACCCGGGGCGCCGC
Rat            TACGCCCTCAAGCCCGACGCCTACCCCGGGCGCCGC
Opossum        ------------------------------------
Platypus       TACGCGCTCAAGCCGGACGCCTACCCGGGCCGCC--
Chick          TACGGCCTCAAACCCGACGCCTTTGCCGGGAGACGC
```

26. C10

```
Human           GCGAAAGAGGAGATAAAGGCAGAAAACACCACAGGAAATTGGCTGACAGCAAAGAGCGGA
Chimp           GCGAAAGAGGAGATAAAGGCAGAAAACACCACAGGAAATTGGCTGACAGCAAAGAGCGGA
Macaque         GCGAAAGAGGAGATAAAGGCAGAAAACACCACAGGAAATTGGCTGACAGCAAAGAGCGGA
Dog             GCGAAAGAGGAGATAAAGGCAGAAAACACCACAGGAAATTGGCTGACAGCAAAGAGCGGA
Cow             GCAAAAGAGGAGATAAAGGCAGAAAACACCACAGGAAATTGGCTGACAGCAAAGAGCGGA
Mouse           GCTAAAGAGGAGATAAAGGCAGAAAACACCACAGGAAATTGGCTGACAGCAAAGAGCGGA
Rat             GCTAAAGAGGAGATAAAGGCAGAAAACACCACAGGAAATTGGCTGACAGCAAAGAGCGGA
Opossum         -------AGGAAATCAAGTCCGATACTCCAACCAGCAATTGGTTAACTGCAAAGAGTGGC
Platypus        ------GAGGACATAAAGGCAGAAAATACTACAGGGAACTGGCTGACAGCGAAGAGCGGA
Chick           ACTCAGAACGATATAAAGGCAGAAAACACTACAGGAAATTGGCTGACAGCAAAGAGCGGA
                       * ** ** *** * ** *   * **  * ** *** * ** ** ***** **

Human           AGGAAGAAGAGGTGCCCCTATACTAAACACCAGACGCTGGAATTGGAGAAAGAATTTCTG
Chimp           AGGAAGAAGAGGTGCCCCTATACTAAACACCAGACGCTGGAATTGGAGAAAGAATTTCTG
Macaque         AGGAAGAAGAGGTGCCCCTATACTAAACACCAGACGCTGGAATTGGAGAAAGAATTTCTG
Dog             AGGAAGAAGAGGTGCCCCTATACTAAACACCAGACGCTGGAATTGGAGAAAGAATTTCTG
Cow             AGGAAGAAGAGGTGCCCCTATACTAAACACCAGACGCTGGAATTGGAGAAAGAATTTCTG
Mouse           AGGAAGAAGAGGTGCCCCTATACTAAACACCAGACGCTGGAATTGGAGAAAGAATTTCTG
Rat             AGGAAGAAGAGGTGCCCCTATACTAAACACCAGACGCTGGAATTGGAGAAAGAATTTCTG
Opossum         AGAAAGAAGAGGTGTCCTTATACTAAACATCAAACACTGGAATTAGAAAAAGAGTTCTTA
Platypus        AGAAAGAAAAGATGTCCTTACACCAAGCACCAGACACTGGAACTGGAGAAGGAATTTCTG
Chick           AGAAAGAAAAGGTGTCCTTACACCAAGCACCAGACGCTGGAGCTGGAGAAGGAATTCTTG
                ** ***** ** ** ** ** ** ** ** ** ** *****  * ** ** ** **  *

Human           TTCAATATGTATTTGACGCGAGAGCGCCGCCTGGAGATTAGCAAGACCATTAACCTTACA
Chimp           TTCAATATGTATTTGACGCGAGAGCGCCGCCTGGAGATTAGCAAGACCATTAACCTTACA
Macaque         TTCAATATGTATTTGACGCGAGAGCGCCGCCTGGAGATTAGCAAGACCATTAACCTTACA
Dog             TTCAATATGTATTTGACGCGAGAGCGCCGCCTGGAGATTAGCAAGACCATTAACCTTACA
Cow             TTCAATATGTATTTGACGCGAGAGCGCCGCCTGGAGATTAGCAAGACCATTAACCTTACA
Mouse           TTCAATATGTATTTGACGCGAGAGCGCCGCCTGGAGATTAGCAAGACCATTAACCTTACA
Rat             TTCAATATGTATTTGACGCGAGAGCGCCGCCTGGAGATTAGCAAGACCATTAACCTTACA
Opossum         TTCAATATGTACCTCACTCGCGAGCGCCGCCTAGAGATCAGTAAGAGCGTTAACCTCACT
Platypus        TTCAATATGTATCTGACGCGAGAGCGCCGCCTGGAGATTAGCAAGAGTATTAACCTGACG
Chick           TTCAATATGTACTTGACCCGTGAGCGCCGCCTGGAGATTAGTAAGAGCATTAACCTGACA
                ***********  * ** ** *********** ***** ** ****   ******* **

Human           GACAGACAAGTCAAAATCTGGTTTCAAAATCGCAGAATGAAACTCAAGAAAATGAACCGA
Chimp           GACAGACAAGTCAAAATCTGGTTTCAAAATCGCAGAATGAAACTCAAGAAAATGAACCGA
Macaque         GACAGACAAGTCAAAATCTGGTTTCAAAATCGCAGAATGAAACTCAAGAAAATGAACCGA
Dog             GACAGACAAGTCAAAATCTGGTTTCAAAATCGCAGAATGAAACTCAAGAAAATGAACCGA
Cow             GACAGACAAGTCAAAATCTGGTTTCAAAATCGCAGAATGAAACTCAAGAAAATGAACCGA
Mouse           GACAGACAAGTCAAAATCTGGTTTCAAAATCGCAGAATGAAACTCAAGAAAATGAACCGA
Rat             GACAGACAAGTCAAAATCTGGTTTCAAAATCGCAGAATGAAACTCAAGAAAATGAACCGA
Opossum         GACAGGCAGGTCAAGATCTGGTTTCAAAACCGCAGAATGAAGCTTAAGAAGATGAGCAGG
Platypus        GACAGACAAGTCAAAATCTGGTTTCAGAACCGAAGGATGAAACTGAAGAAAATGAACCGG
Chick           GACAGACAAGTCAAAATCTGGTTTCAGAACCGCAGGATGAAACTCAAGAAAATGAACAGA
                ***** ** ***** *********** ** ** ** ***** ** ***** **** * *

Human           GAGAATCGGATCCGGGAACTGACCTCCAATTTTAATTTCACC
Chimp           GAGAATCGGATCCGGGAACTGACCTCCAATTTTAATTTCACC
Macaque         GAGAATCGGATCCGGGAACTGACCTCCAATTTTAATTTCACC
Dog             GAGAATCGGATCCGGGAACTGACCTCCAATTTTAATTTCACC
Cow             GAGAATCGGATCCGGGAACTGACCTCCAATTTTAATTTCACC
Mouse           GAGAATCGGATCCGGGAACTGACCTCCAATTTTAATTTCACC
Rat             GAGAATCGGATCCGGGAACTGACCTCCAATTTTAATTTCACC
Opossum         GAGAACCGAATCCGAGAACTGACCGCTAATCTGACCTTCTCT
Platypus        GAGAACCGGATTCGCGAACTGACCTCCAATTTCAGTTTCACC
Chick           GAGAACCGAATCCGCGAACTGACGTCCAATTTTAATTTCACC
                ***** ** ** ** ********  * *** * *  *** *
```

27. C11

```
Human           GGAGCCGCC---------CCCAACGCCCCCCGCACCCGCAAGAAGCGCTGCCCTTATTCG
Chimp           GGAGCCGCC---------CCCAACGCCCCCCGCACCCGCAAGAAGCGCTGCCCTTATTCG
Macaque         GGAGGGGCCCGAGGGCACGCACACGCCCCCCGCACCCGCAAGAAGCGCTGCCCTTATTCG
Dog             GGAGCCGCC---------CCC---------------------------------------
Cow             GGAGCCGCC---------CCCAACGCCCCCCGCACCCGCAAGAAGCGCTGCCCTTATTCG
Mouse           GGAGCCGCC---------CCCAACGCCCCCCGCACCCGCAAGAAGCGCTGCCCTTATTCG
Rat             GGAGCCGCC---------CCCAACGCCCCCCGAACCCGCAAGAAGCGCTGCCCTTATTCG
Opossum         ------------------------------------------------------------
Platypus        ----------------------ACGCCCCCCGCGCTCGGAAGAAACGCTGTCCTTACTCC
Chick           ------------------TCGAGTGCTCCCCGAACGAGGAAGAAGAGGTGCCCCTATTCG


Human           AAATTCCAGATCCGGGAACTGGAGCGAGAGTTTTTCTTCAACGTGTATATCAACAAAGAG
Chimp           AAATTCCAGATCCGGGAACTGGAGCGAGAGTTTTTCTTCAACGTGTATATCAACAAAGAG
Macaque         AAATTCCAGATCCGGGAACTGGAGCGAGAGTTTTTCTTCAACGTATATATCAACAAAGAG
Dog             ---------------------GAGCGAGAGTTTTTCTTCAACGTGTATATCAACAAAGAG
Cow             AAATTCCAGATCCGGGAACTGGAGCGAGAGTTTTTCTTCAACGTGTATATCAACAAAGAG
Mouse           AAATTCCAGATCCGGGAACTGGAGCGAGAGTTTTTCTTCAACGTGTACATCAACAAAGAG
Rat             AAATTCCAGATCCGGGAACTGGAGCGAGAGTTTTTCTTCAACGTGTACATCAACAAAGAG
Opossum         ------------------------------------------------------------
Platypus        AAGTTTCAGATCCGGGAACTGGAGCGGGAGTTTTTCTTCAACGTCTACATCAACAAAGAG
Chick           AAATTCCAGATCAGAGAGCTGGAGAGAGAATTCTTCTTCAATGTCTATATAAACAAAGAA


Human           AAGCGGCTGCAGCTGTCCCGGATGCTGAACCTGACGGACCGACAAGTGAAAATTTGGTTT
Chimp           AAGCGGCTGCAGCTGTCCCGGATGCTGAACCTGACGGACCGACAAGTGAAAATTTGGTTT
Macaque         AAGCGGCTGCAGCTGTCCCGGATGCTGAACCTGACGGACCGACAAGTGAAAATTTGGTTT
Dog             AAGCGGCTGCAGCTGTCCCGGATGCTGAACCTGACGGACCGACAAGTGAAAATTTGGTTT
Cow             AAGCGGCTGCAGCTGTCTCGGATGCTGAACCTGACGGACCGACAAGTGAAAATTTGGTTT
Mouse           AAGCGGCTGCAGCTGTCCCGGATGCTGAACCTGACAGACCGACAAGTGAAAATTTGGTTT
Rat             AAGCGGCTGCAGCTGTCCCGCATGCTGAACCTGACAGACCGACAAGTGAAAATTTGGTTT
Opossum         ------------------------------------------------------------
Platypus        AAGAGACTGCAACTGTCCAGGATGCTAAACCTGACAGACCGACAAGTGAAAATTTGGTTT
Chick           AAGAGGCTCCAGTTGTCTCGGATGCTGAATCTCACTGACCGACAAGTTAAAATCTGGTTT


Human           CAGAACAGAAGGATGAAAGAA
Chimp           CAGAACAGAAGGATGAAAGAA
Macaque         CAGAACAGAAGGATGAAAGAA
Dog             CAGAACAGAAGGATGAAAGAA
Cow             CAGAACAGAAGGATGAAAGAA
Mouse           CAGAACAGGAGAATGAAAGAA
Rat             CAGAACAGAAGAATGAAAGAA
Opossum         ---------------------
Platypus        CAGAACAGAAGGATGAAAGAG
Chick           CAGAACAGAAGAATGAAAGAA
```

28. C12

```
Human           CAGCGCCGGAGGGAACTCTCAGACCGCTTGAATCTTAGTGACCAGCAGGTCAAGATCTGG
Chimp           CAGCGCCGGAGGGAACTCTCAGACCGCTTGAATCTTAGTGACCAGCAGGTCAAGATTTGG
Macaque         CAGCGCCGGAGGGAACTCTCAGACCGCTTGAATCTTAGTGACCAGCAGGTCAAGATCTGG
Dog             CAGCGCCGGAGGGAACTCTCAGACCGCTTGAATCTTAGTGACCAGCAGGTCAAGATCTGG
Cow             CAGCGCCGGAGGGAACTCTCAGACCGCTTGAATCTTAGTGACCAGCAGGTCAAGATCTGG
Mouse           CAGCGTCGGAGGGAACTCTCGGACCGCTTGAATCTTAGTGATCAGCAGGTCAAGATTTGG
Rat             CAGCGTCGGAGGGAACTCTCGGACCGCTTGAATCTTAGTGATCAGCAGGTCAAGATTTGG
Opossum         ------------------------------------------------------------
Platypus        ------------------------------------------------------------
Chick           CAGAAGAGGAAAGAACTATCAAACAGACTGAATTTAAGCGACCAGCAAGTGAAAATTTGG


Human           TTTCAGAACCGGAGAATGAAAAAGAAAAGACTTCTGTTGAGGGAGCAAGCTCTCTCCTTC
Chimp           TTTCAGAACCGGAGAATGAAAAAGAAAAGACTTCTGTTGAGGGAGCAAGCTCTCTCCTTC
Macaque         TTTCAGAACCGGAGAATGAAAAAGAAAAGACTTCTGTTGAGGGAGCAAGCTCTCTCCTTC
Dog             TTTCAGAACCGGAGAATGAAAAAGAAAAGACTTCTGTTGAGGGAGCAAGCTCTCTCCTTC
Cow             TTTCAGAACCGCAGAATGAAAAAGAAAAGACTTCTGTTGAGGGAGCAAGCTCTCTCCTTC
Mouse           TTCCAGAACCGGAGAATGAAAAAGAAAAGACTTCTGCTGAGGGAGCAAGCTCTCTCCTTC
Rat             TTCCAGAACCGGAGAATGAAAAAGAAAAGACTTCTGCTGAGAGAGCAAGCTCTCTCCTTC
Opossum         ------------------------------------------------------------
Platypus        ------------------------------------------------------------
Chick           TTTCAGAACCGACGAATGAAAAAGAAAAGAGTGGTAATGCGCGAGCAGGCGCTCTCTATG


Human           TTT
Chimp           TTT
Macaque         TTT
Dog             TTT
Cow             TTT
Mouse           TTC
Rat             TTC
Opossum         ---
Platypus        ---
Chick           TAC
```

29. D8

```
Human           CCTCCCCCCTGCGGCGGGATTGCCTGTCACGGGGAGCCCGCGAAGTTTTACGGATACGAT
Chimp           CCTCCCCCCTGCGGCGGGATTGCCTGTCACGGGGAGCCCGCGAAGTTTTACGGATACGAT
Macaque         CCTCCCCCCTGCGGCGGGATTGCCTGTCACGGGGAGCCCGCGAAGTTTTACGGATACGAT
Dog             CCTCCCCCCTGCGGCGGGATTGCCTGTCACGGGGAGCCCGCGAAGTTTTACGGATACGAT
Cow             CCTCCCCCCTGCGGTGGGATTGCCTGTCACGGGGAGCCCGCGAAGTTTTACGGATACGAT
Mouse           CCTCCCCCCTGCGGCGGGATTGCCTGTCACGGGGAGCCCGCGAAGTTTTACGGATACGAT
Rat             CCTCCCCCCTGCGGCGGGATTGCCTGTCACGGGGAGCCCGCGAAGTTTTACGGATACGAT
Opossum         CCTCCCCCCTGCAGTGGGATTGCCTGTCACGGGGAGCCCGCTAAGTTTTACGGATACGAT
Platypus        CCTCCCCCCTGCAGCGGGATTGCCTGTCACGGGGAGCCCGCTAAATTTTACGGATACGAT
Chick           ------CCCTGCAGCGGGGTTACCTGTCACGGGGAGCCCGCTAAATTTTACGGATACGAT
                      ****** * *** ** ******************* ** ***************

Human           AACTTACAGAGACAGCCGATTTTTACGACCCAGCAAGAGGCCGAGCTGGTACAATATCCT
Chimp           AACTTACAGAGACAGCCGATTTTTACGACCCAGCAAGAGGCCGAGCTGGTACAATATCCT
Macaque         AACTTACAGAGACAGCCGATTTTTACGACCCAGCAAGAGGCCGAGCTGGTACAATATCCT
Dog             AACTTACAGAGACAGCCGATTTTTACGACCCAGCAAGAGGCCGAGCTGGTACAATATCCT
Cow             AACTTACAGAGACAGCCGATTTTTACGACCCAGCAAGAGGCCGAGCTGGTACAATATCCT
Mouse           AACTTACAGAGACAGCCGATTTTTACGACCCAGCAAGAGGCCGAGCTGGTACAATATCCT
Rat             AACTTACAGAGACAGCCGATTTTTACGACCCAGCAAGAGGCCGAGCTGGTACAATATCCT
Opossum         AACTTACAGAGACAGCCGATTTTTACGACCCAGCAAGAGGCCGAGCTGGTACAATATCCT
Platypus        AACTTACAGAGACAGCCGATTTTTACGACCCAGCGAGAGGCCGAGCTGGTACAATATCCT
Chick           AACTTACAGAGACAGCAGATTTTTACGACACAGCAAGAGGCCGAGCTGGTACAATATCCT
                **************** ************ **** *************************

Human           GACTGTAAATCGTCCAGTGGTAATATTGGCGAGGACCCAGACCACTTAAATCAGAGCTCG
Chimp           GACTGTAAATCGTCCAGTGGTAATATTGGCGAGGACCCAGACCACTTAAATCAGAGCTCG
Macaque         GACTGTAAATCGTCCAGTGGTAATATTGGCGAGGACCCAGACCACTTAAATCAGAGCTCG
Dog             GACTGTAAATCGTCCAGTGGTAATATTGGCGAGGACCCAGACCACTTAAATCAGAGCTCG
Cow             GACTGTAAATCGTCCAGTGGTAATATTGGCGAGGACCCAGACCACTTAAATCAGAGCTCG
Mouse           GACTGTAAATCGTCCAGTGGTAATATTGGCGAGGACCCAGACCACTTAAATCAGAGCTCG
Rat             GACTGTAAATCGTCCAGTGGTAATATTGGCGAGGACCCAGACCACTTAAATCAGAGCTCG
Opossum         GACTGTAAATCGTCCAGTGGTAATATTGGCGAGGACCCAGACCACTTAAATCAGAGCTCG
Platypus        GACTGTAAATCGTCCAGTGGTAATATTGGCGAAGACCCAGACCACTTAAATCAGAGCTCG
Chick           GACTGTAAATCGTCCAGTGCTAATATTGGCGAGGAACCAGACCACTTAAATCAGAGCTCG
                ******************* ************ ** ************************

Human           TCTCCTTCTCAAATGTTT
Chimp           TCTCCTTCTCAAATGTTT
Macaque         TCTCCTTCTCAAATGTTT
Dog             TCTCCTTCTCAAATGTTT
Cow             TCTCCTTCTCAAATGTTT
Mouse           TCTCCTTCTCAAATGTTT
Rat             TCTCCTTCTCAAATGTTT
Opossum         TCTCCTTCTCAAATGTTT
Platypus        TCTCCTTCTCAAATGTTT
Chick           TCTCCCGCTCAAATGTTT
                *****  ***********
```

30. D10-1

```
Human           ATGTCCTTTCCCAACAGCTCTCCTGCTGCTAATACTTTTTTAGTAGATTCCTTGATCAGT
Chimp           ATGTCCTTTCCCAACAGCTCTCCTGCTGCTAATACTTTTTTAGTAGATTCCTTGATCAGT
Macaque         ATGTCCTTTCCCAACAGCTCTCCTGCTGCTAATACTTTTTTAGTAGATTCCTTGATCAGT
Dog             ATGTCCTTTCCCAACAGCTCTCCTGCTGCTAATACTTTTTTAGTAGATTCCTTGATCAGT
Cow             ATGTCCTTTCCCAACAGCTCTCCTGCTGCTAATACTTTTTTAGTAGATTCCTTGATCAGT
Mouse           ATGTCCTTTCCCAACAGCTCTCCTGCTGCTAATACTTTTTTAGTAGATTCCTTGATCAGT
Rat             ATGTCCTTTCCCAACAGCTCTCCTGCTGCTAATACTTTTTTAGTAGATTCCTTGATCAGT
Opossum         ATGTCCTTTCCCAACAGCTCTCCTGCTGCTAATACTTTTTTAGTAGATTCCTTGATCAGT
Platypus        ATGTCGTTTCCCAACAGCTCTCCTGCTGCTAATACTTTTTTAGTAGATTCCTTGATCAGT
Chick           ATGTCCTTTCCCAACAGCTCTCCTGCCGCTAATACTTTTTTAGTAGATTCTCTCATCAGT
                ***** ******************** ***********************  * ******

Human           GCCTGCAGGAGTGACAGTTTTTATTCCAGCAGCGCCAGCATGTACATGCCACCACCTAGC
Chimp           GCCTGCAGGAGTGACAGTTTTTATTCCAGCAGCGCCAGCATGTACATGCCACCACCTAGC
Macaque         GCCTGCAGGAGTGACAGTTTTTATTCGAGCAGCGCCAGCATGTACATGCCACCACCTAGC
Dog             GCCTGCAGGAGTGACAGTTTTTATTCGAGCAGCGCCAGCATGTACATGCCACCACCTAGC
Cow             GCCTGCAGGAGTGACAGTTTTTATTCGAGCAGCGCCAGCATGTACATGCCACCACCTAGC
Mouse           GCCTGCAGGAGTGACAGTTTTTATTCGAGCAGCGCCAGCATGTACATGCCACCACCTAGC
Rat             GCCTGCAGGAGTGACAGTTTTTATTCGAGCAGCGCCAGCATGTACATGCCACCACCTAGC
Opossum         GCCTGCAGGAGTGACAGTTTTTATTCTAGCAGCGCCAGCATGTACATGCCACCACCCAGC
Platypus        GCCTGTAGGAGTGACAGTTTTTATTCTAGCAGCGCCAGCATGTATATGCCAACACCTAAC
Chick           GCGTGCAGGAGTGACAGTTTCTACTCCAACAGCGCCAGCATGTATATGCCA---CCTAGC
                ** ** ************** ** ** * *************** ******   ** * *

Human           GCAGACATGGGGACCTATGGAATGCAAACCTGTGGACTGCTCCCGTCTCTGGCCAAAAGA
Chimp           GCAGACATGGGGACTTATGGAATGCAAACCTGTGGACTGCTCCCGTCTCTGGCCAAAAGA
Macaque         GCAGACATGGGGACCTATGGAATGCAAACCTGTGGACTGCTCCCGTCTCTGGCCAAAAGA
Dog             GCAGACATGGGGACCTATGGAATGCAAACCTGTGGACTGCTCCCGTCTCTGGCCAAAAGA
Cow             GCAGACATGGGGACCTATGGAATGCAAACCTGTGGACTGCTCCCGTCTCTGGCCAAAAGA
Mouse           GCAGACATGGGGACCTATGGAATGCAAACCTGTGGACTGCTCCCGTCTTTGGCCAAAAGA
Rat             GCAGACATGGGGACCTATGGAATGCAAACCTGTGGACTGCTCCCGTCTCTGGCCAAAAGA
Opossum         ACAGACATGGGGACTTATGGAATGCAAACCTGTGGACTGCTCCCGTCTCTGGCTAAAAGA
Platypus        ACAGACATGGGGACTTATGGAATGCAAACCTGTGGACTGCTACCGTCTCTGGCTAAAAGA
Chick           ACAGACATTGGGACTTATGGGATGCAAACCTGTGGACTCCTACCGTCTCTGGCTAAAAGA
                 ******* ***** ***** ***************** ** ****** **** ******

Human           GAAGTGAACCACCAAAATATGGGTATGAATGTGCATCCTTATATACCTCAAGTAGACAGT
Chimp           GAAGTGAACCACCAAAATATGGGTATGAATGTGCATCCTTATATACCTCAAGTAGACAGT
Macaque         GAAGTGAACCACCAAAATATGGGTATGAATGTGCATCCTTATATACCTCAAGTAGACAGT
Dog             GAAGTGAACCACCAAAATATGGGTATGAATGTGCATCCTTATATACCTCAAGTAGACAGT
Cow             GAAGTGAACCACCAAAATATGGGTATGAATGTGCATCCTTATATACCTCAAGTAGACAGT
Mouse           GAAGTGAACCACCAAAATATGGGTATGAATGTACATCCTTATATACCTCAAGTAGACAGT
Rat             GAAGTGAACCACCAAAATATGGGTATGAATGTACATCCTTATATACCTCAAGTAGACAGT
Opossum         GAAGTTAATCACCAAAATATGGGTATGAATGTGCATCCTTATATACCTCAAGTAGAGAGT
Platypus        GAAGTTAATCACCAAAATATGGGTATGAATGTACATCCTTATATACCTCAAGTAGACAGT
Chick           GAAGTTAATCACCAAAATATGGGTATGAATGTACATCCTTATATACCTCAAGTAGACAGT
                ***** ** *********************** *********************** ***

Human           TGGACA
Chimp           TGGACA
Macaque         TGGACA
Dog             TGGACA
Cow             TGGACA
Mouse           TGGACA
Rat             TGGACA
Opossum         TGGACA
Platypus        TGGACA
Chick           TGGACA
                ******
```

31. D10-2

```
Human           CCGAACAGATCTTGTCGAATAGAGCAACCTGTTACACAGCAAGTCCCCACTTGCTCCTTC
Chimp           CCGAACAGATCTTGTCGAATAGAGCAACCTGTTACACAGCAAGTCCCCACTTGCTCCTTC
Macaque         CCGAACAGATCTTGTCGAATAGAGCAACCTGTTACACAGCAAGTCCCCACTTGCTCCTTC
Dog             CCGAACAGATCTTGTCGAATAGAGCAACCTGTTACACAGCAAGTCCCCACTTGCTCCTTC
Cow             CCGAACAGATCTTGTCGAATAGAGCAACCTGTTACACAGCAAGTCCCCACTTGCTCCTTC
Mouse           CCGAACAGATCTTGTCGAATAGAGCAACCTGTTACACAGCAAGTCCCCACTTGCTCATTC
Rat             CCGAACAGATCTTGTCGAATAGAGCAACCTGTTACACAGCAAGTCCCCACTTGCTCATTC
Opossum         CCGAGCAGATCTTGTCGAATAGAGCAACCTGTTACACAGCAAGTCCCTACTTGCTCCTTC
Platypus        GCGAACAGGTCCTGCCGAATAGAGCAACCTGTTACACAGCAAGTACCCACTTGCTCCTTC
Chick           CCGAACAGATCTTGTCGAATAGAGCAACCTGTTACACAGCAGGTCCCCACTTGTTCCTTC
                 *** *** ** ** ************************** ** ** ***** ** ***

Human           ACCACCAACATTAAGGAAGAATCCAATTGCTGCATGTATTCTGATAAGCGCAACAAACTC
Chimp           ACCACCAACATTAAGGAAGAATCCAATTGCTGCATGTATTCTGATAAGCGCAACAAACTC
Macaque         ACCACCAACATTAAGGAAGAATCC------------------------------------
Dog             ACCACCAACATTAAGGAAGAATCCAATTGCTGCATGTATTCTGATAAGCGCAACAAACTC
Cow             ACCACCAACATTAAGGAAGAATCCAATTGCTGCATGTATTCTGATAAACGCAACAAACTC
Mouse           ACCGCCAACATTAAAGAAGAATCCAATTGCTGCATGTATTCTGATAAGCGCAACAAACTC
Rat             ACCGCCAACATTAAAGAAGAATCCAATTGCTGCATGTATTCTGATAAGCGCAACAAACTC
Opossum         ACTACCAATATTAAGGAAGAAACCAATTGCTGCATGTATTCTGATAAGAGAACCAAACTC
Platypus        ACCACCAACATTAAGGAAGAAACCAATTGCTGCATGTATTCCGATAAGCGAACCAAACTC
Chick           ACTACAAATATTAAGGAAGAAAGCAATTGCTGCATGTATTCCGATAAGAGAACCAAACTC
                **  * ** ***** ******  *

Human           ATT
Chimp           ATT
Macaque         ---
Dog             ATT
Cow             ATT
Mouse           ATT
Rat             ATT
Opossum         ATT
Platypus        GTT
Chick           ATT
```

32. D10-3

```
Human       CCAACCAGCAATTGGCTCACTGCAAAGAGTGGCAGAAAGAAGAGGTGCCCTTACACTAAG
Chimp       CCAACCAGCAATTGGCTCACTGCAAAGAGTGGCAGAAAGAAGAGGTGCCCTTACACTAAG
Macaque     ------------------------------------------------------------
Dog         CCAACCAGCAATTGGCTCACTGCAAAGAGTGGCAGAAAGAAGAGGTGCCCTTACACTAAG
Cow         CCAACCAGCAATTGGCTCACTGCAAAGAGTGGCAGAAAGAAGAGGTGCCCTTACACTAAG
Mouse       CCAACCAGCAATTGGCTCACTGCAAAGAGTGGCAGAAAGAAGAGGTGCCCTTACACCAAG
Rat         CCAACCAGCAATTGGCTCACTGCAAAGAGTGGCAGAAAGAAGAGGTGCCCTTACACTAAA
Opossum     CCAACCAGCAATTGGTTAACTGCAAAGAGTGGCAGAAAGAAGAGGTGTCCTTATACTAAA
Platypus    ------------------------------------------------------------
Chick       ACTACAGGAAATTGGCTGACAGCAAAGAGCGGAAGAAAGAAAAGGTGTCCTTACACCAAG


Human       CACCAAACGCTGGAATTAGAAAAAGAGTTCTTGTTCAATATGTACCTCACCCGCGAGCGC
Chimp       CACCAAACGCTGGAATTAGAAAAAGAGTTCTTGTTCAATATGTACCTCACCCGCGAGCGC
Macaque     ------------------------------------------------------------
Dog         CACCAAACGCTGGAATTAGAAAAAGAGTTCTTGTTCAATATGTACCTCACCCGCGAGCGC
Cow         CACCAAACGCTGGAATTAGAAAAAGAGTTCTTGTTCAATATGTACCTCACCCGCGAGCGC
Mouse       CACCAAACGCTGGAATTGGAAAAAGAGTTCTTGTTCAATATGTACCTCACCCGCGAGCGC
Rat         CACCAAACGCTGGAATTAGAAAAAGAGTTCTTGTTCAATATGTACCTCACCCGCGAGCGC
Opossum     CATCAAACACTGGAATTAGAAAAAGAGTTCTTATTCAATATGTACCTCACTCGCGAGCGC
Platypus    ------------------------------------------------------------
Chick       CACCAGACGCTGGAGCTGGAGAAGGAATTCTTGTTCAATATGTACTTGACCCGTGAGCGC


Human       CGCCTAGAGATCAGTAAGAGCGTTAACCTCACCGACAGGCAGGTCAAGATTTGGTTTCAA
Chimp       CGCCTAGAGATCAGTAAGAGCGTTAACCTCACCGACAGGCAGGTCAAGATTTGGTTTCAA
Macaque     ------------------------------------------------------------
Dog         CGCCTAGAGATCAGTAAGAGCGTTAACCTCACCGACAGGCAGGTCAAGATTTGGTTTCAA
Cow         CGCCTAGAGATCAGTAAGAGCGTTAACCTCACCGACAGGCAGGTCAAGATTTGGTTTCAA
Mouse       CGCCTAGAGATCAGTAAGAGCGTTAACCTCACCGACAGGCAGGTCAAGATTTGGTTTCAA
Rat         CGCCTAGAGATCAGTAAGAGCGTTAACCTCACCGACAGGCAGGTCAAGATTTGGTTTCAA
Opossum     CGCCTAGAGATCAGTAAGAGCGTTAACCTCACTGACAGGCAGGTCAAGATCTGGTTTCAA
Platypus    ------------------------------------------------------------
Chick       CGCCTGGAGATTAGTAAGAGCATTAACCTGACAGACAGACAAGTCAAAATCTGGTTTCAG


Human       AACCGCCGAATGAAACTCAAGAAGATGAGCCGAGAGAACCGGATCCGAGAACTGACCGCC
Chimp       AACCGCCGAATGAAACTCAAGAAGATGAGCCGAGAGAACCGGATCCGAGAACTGACCGCC
Macaque     ------------------------------------------------------------
Dog         AACCGCCGAATGAAACTCAAGAAGATGAGCCGAGAGAACCGGATCCGAGAACTGACCGCC
Cow         AACCGCCGAATGAAACTCAAGAAGATGAGCCGAGAGAACCGGATCCGAGAACTGACCGCC
Mouse       AACCGCCGAATGAAACTCAAGAAGATGAGCCGAGAGAATCGAATCCGAGAACTGACCGCC
Rat         AACCGCCGAATGAAACTCAAGAAGATGAGCCGAGAGAATCGAATCCGAGAACTGACCGCC
Opossum     AACCGCAGAATGAAGCTTAAGAAGATGAGCAGGGAGAACCGAATCCGAGAACTGACCGCT
Platypus    ------------------------------------------------------------
Chick       AACCGCAGGATGAAACTCAAGAAAATGAACAGAGAGAACCGAATCCGCGAACTGACGTCC


Human       AACCTC
Chimp       AACCTC
Macaque     ------
Dog         AACCTC
Cow         AACCTC
Mouse       AACCTC
Rat         AACCTC
Opossum     AATCTG
Platypus    ------
Chick       AATTTT
```
