# Supplementary figures and images for: Ultraconserved coding regions outside the homeobox of mammalian Hox genes
Source: BMC Evol Biol. 2008 Sep 24;8:260. doi: 10.1186/1471-2148-8-260 (PMC2566984; doi:10.1186/1471-2148-8-260)

## Additional file 4

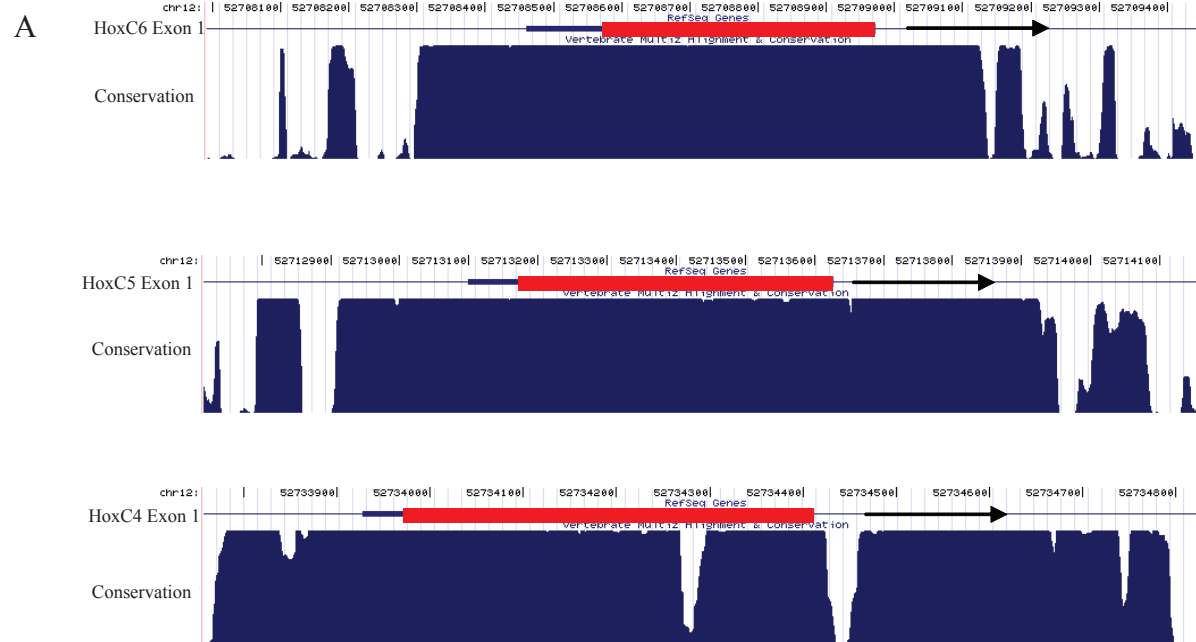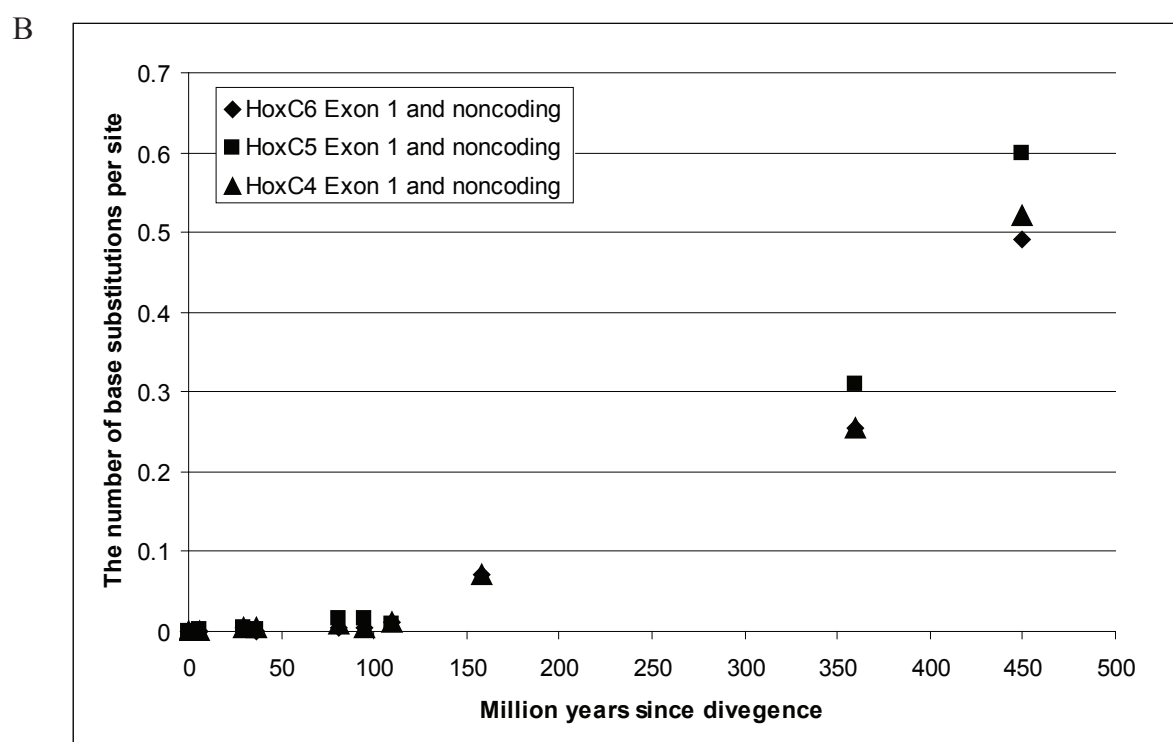

Supplement: Additional file 4 — Conserved noncoding sequences flanking the UCRs. A. The conservation of noncoding sequences flanking the first exons of HoxC4, HoxC5 and HoxC6 from UCSC Human Genome Brower. The position of the coding regions is highlighted by red bar. Transcription direction is indicated by arrow. B. The accumulations of nucleotide mutations in the conserved regions with divergence times of the three Hox genes. The number of substitution per site was estimated using the Jukes-Cantor's method in MEGA4 on the coding region and flanking conserved noncoding sequences of HoxC4, HoxC5 and HoxC6. [file 1471-2148-8-260-S4.pdf]
